# Ubiquitin specific *peptidase Usp53* regulates osteoblast versus adipocyte lineage commitment

**DOI:** 10.1038/s41598-021-87608-x

**Published:** 2021-04-19

**Authors:** Hadla Hariri, William N. Addison, René St-Arnaud

**Affiliations:** 1 Research Centre, Shriners Hospital for Children – Canada, 1003 Decarie Boulevard, Montreal, QC H4A 0A9 Canada; 2 Department of Human Genetics, McGill University, Montreal, QC Canada; 3 Department of Surgery, McGill University, Montreal, QC Canada; 4 Department of Medicine, McGill University, Montreal, QC Canada

**Keywords:** Cell biology, Molecular biology

## Abstract

We have previously shown that parathyroid hormone (PTH) induces the phosphorylation of the DNA-binding protein Nascent polypeptide associated complex And Coregulator alpha (NACA), leading to nuclear translocation of NACA and activation of target genes. Using ChIP-Seq against NACA in parallel with RNA-sequencing, we report the identification of *Ubiquitin Specific Peptidase 53 (Usp53)* as a target gene of PTH-activated NACA in osteoblasts. A binding site for NACA within the ChIP fragment from the *Usp53* promoter was confirmed by electrophoretic mobility shift assay. Activity of the *Usp53* promoter (− 2325/+ 238 bp) was regulated by the JUN-CREB complex and this activation relied on activated PKA and the presence of NACA. *Usp53* knockdown in ST2 stromal cells stimulated expression of the osteoblastic markers *Bglap2* (Osteocalcin) and *Alpl* (Alkaline phosphatase) and inhibited expression of the adipogenic markers *Pparg* and *Cebpa*. A similar effect was measured when knocking down *Naca*. During osteoblastogenesis, the impact of *Usp53* knockdown on PTH responses varied depending on the maturation stage of the cells. In vivo implantation of *Usp53*-knockdown bone marrow stromal cells in immunocompromised mice showed an increase in osteoblast number and a decrease in adipocyte counts. Our data suggest that *Usp53* modulates the fate of mesenchymal cells by impacting lineage selection.

## Introduction

Protein ubiquitination is a reversible post-translational modification that regulates a multitude of biological functions including cell cycle regulation, kinase signaling, protein degradation, and DNA repair^1^. Deubiquitination by deubiquitinating enzymes (DUBs) counter-regulates this event and replenishes the ubiquitin pool. The importance of DUBs has been highlighted in various fields. Several studies have provided insight on how to define the DUBs interaction landscape, their regulation, and physiological roles^2,3^. The human genome encodes around 97 DUBs, of which 79 are predicted to be catalytically active^4^. DUBs cluster in five main families: ubiquitin-C-terminal hydrolases (UCHs), ubiquitin-specific peptidases (USPs), ovarian tumor proteases (OTUs), Machado-Joseph disease protein domain proteases (MJDs), and JAB1/MPN/MOV34 metalloenzymes (JAMMs)^4^.

The largest number of DUBs clusters under the USP umbrella^4^. The genomic landscape encompasses about 58 different USPs, few of which have a clear assigned function and substrate. Based on sequence analysis, six USPs (USP39, USP50, USP52, USP53, USP54, and USPL1) are predicted to be inactive, yet experimental evidence is still scarce^5^. One study has linked *Usp53* to progressive hearing loss in mice and confirmed that USP53 exhibits a catalytically inactive domain, suggesting a mechanism of protein–protein interactions involving USP53^6^. Despite the progress achieved so far in understanding the physiological role of USP53, nothing is known about the transcriptional regulation and function of this gene in bone biology.

Parathyroid Hormone (PTH) stands as a master regulator of mineral homeostasis. Incessant infusion of PTH promotes calcium and phosphorous release from bone through activation of osteoclast-mediated resorption^7,8^. However, PTH functions within its anabolic window through intermittent (iPTH) treatment that promotes bone formation through pleiotropic effects on osteoblasts and osteocytes^9–11^. In the cell, numerous studies have uncovered a multitude of signaling cascades that function downstream of PTH, parallel or synergistically to achieve the full anabolic response of iPTH (reviewed in^12,13^). The work of our group has characterized one of these signal transduction pathways: the PTH-Gαs-PKA-NACA pathway^14^. We have demonstrated the physiological relevance of this pathway in vivo and dissected the cascade that initiates with PTH binding to its receptor, PKA-dependent phosphorylation of the transcriptional coregulator NACA on residue Serine 99, and nuclear translocation of NACA^14^. NACA (alpha chain of the nascent polypeptide-associated complex, αNAC) is a physiologically relevant transcriptional coregulator of osteogenic targets in bone tissue^14,15^. NACA has been found to specifically bind DNA and assemble with transcription factors such as JUN and JUND homodimers to enhance the transcription of the osteocalcin (*Bglap2*) and *Lrp6* genes^15–18^. More recently, we have shown that NACA is required for the CREB-mediated activation of the *Nfil3* gene in osteoblasts^19^.

Here, we report the identification of *Usp53* as a downstream target of the PTH-NACA pathway in osteoblasts, using Chromatin Immunoprecipitation with deep sequencing (ChIP-Seq) against NACA and RNA-Seq in MC3T3-E1 cells treated with PTH(1–34). We describe a mechanism downstream from activated PKA involving the JUN-CREB complex along with NACA regulating transcription from the *Usp53* promoter in osteoblasts. Additionally, we describe a role for USP53 in mesenchymal cell differentiation. We report that the silencing of *Usp53* in ST2 stromal cells enhanced osteoblastogenesis and inhibited adipogenesis in vitro. In addition, *Usp53*-deficient bone marrow stromal cells (BMSCs, also known as bone-marrow derived mesenchymal stem cells) exhibited more bone and less fat when implanted in immunocompromised mice.

## Results

### The PTH-NACA pathway regulates *Usp53* in osteoblasts

Our lab has characterized the binding of NACA to multiple gene promoters in osteoblasts. In one study, the PTH (1–34) stimulation of osteoblasts induces the phosphorylation of NACA at S99, its subsequent nuclear translocation and binding to the *Bglap2* promoter^14^. Another study has highlighted the role of NACA along with JUND in *Lrp6* transcriptional regulation in MC3T3-E1 cells^18^.

**Figure 1:**
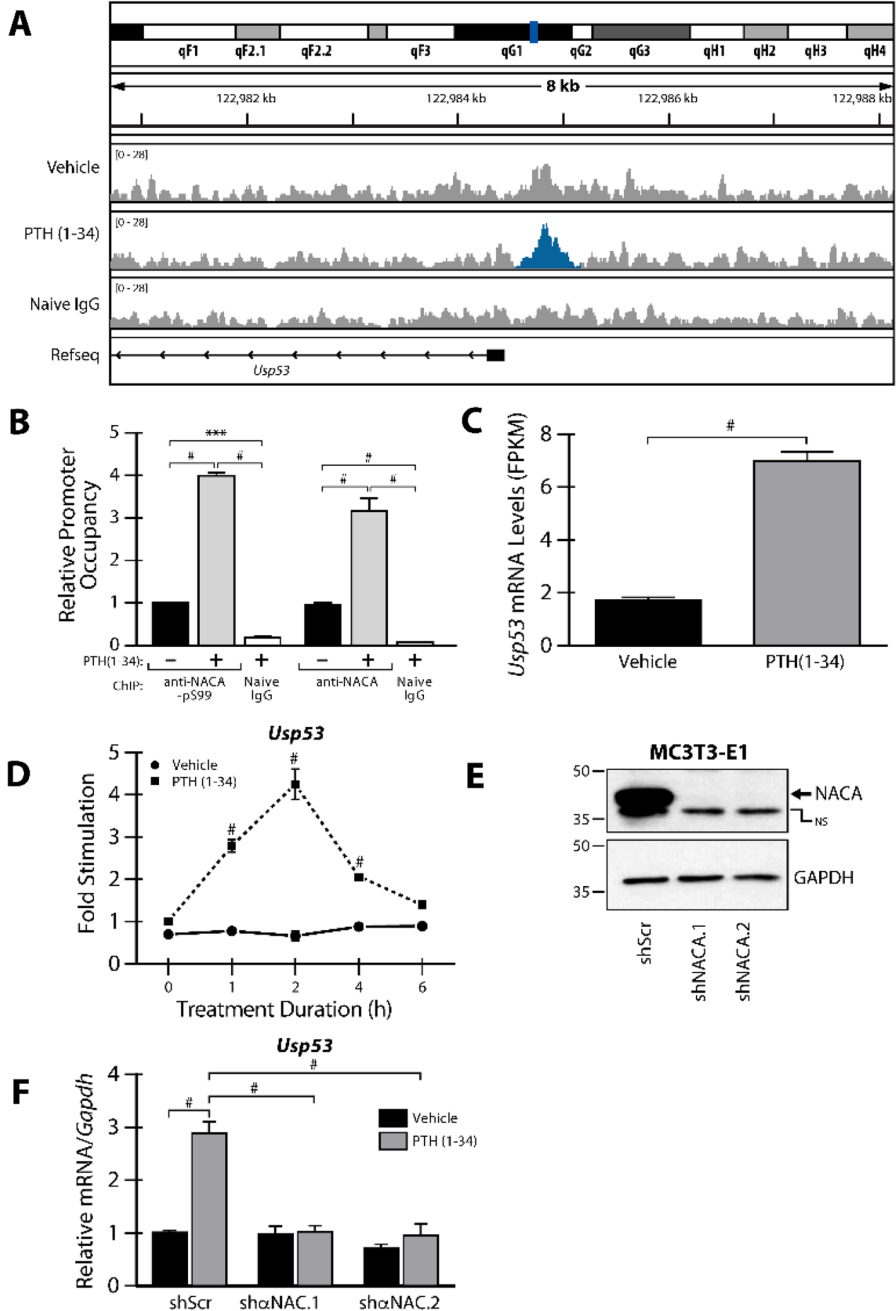
ChIP-Seq and RNA-Seq identify *Usp53* as a target of the PTH-NACA axis in osteoblasts. (**A**) Annotated screenshot given by the Integrative Genomics Viewer (IGV) showing NACA-ChIP-Seq peak (highlighted in blue) detected in the proximal region of the *Usp53* promoter. ChIP-Seq was performed with anti-NACA-pS99 antibody or naïve IgG on MC3T3-E1 cells induced or not with 100 nM PTH(1–34) for 30 min. (**B**) NACA binds the proximal *Usp53* promoter. Quantitative chromatin immunoprecipitation (ChIP) was performed with anti-NACA-pS99-specific antibody, pan-specific anti-NACA antibody, or naive IgG on MC3T3-E1 cells induced or not with 100 nM PTH(1–34) for 30 min. Inputs and bound DNA sequences were amplified by Real Time PCR (RT-PCR) using SYBR green technologies and specific primers flanking the NACA binding site within the *Usp53* promoter. Relative promoter occupancy was calculated as enrichment over vehicle treated cells and results are means ± SD of three independent experiments (n = 3). (**C**) Induction of *Usp53* mRNA expression post treatment by PTH(1–34) for 1 h as determined by RNA-sequencing. Results are shown as fragments per kilobase of exon per million fragments mapped (FPKM), as given by RNA-sequencing. (**D**) RT-qPCR analysis of mRNA expression for *Usp53* in primary osteoblast cells treated with vehicle or 100 nM PTH(1–34) for the indicated time points. (**E**) Immunoblot of whole cells extracts from MC3T3-E1 cells stably expressing shRNAs targeting *Naca* (shNACA.1 and shNACA.2) and scrambled shRNA (shScr) as control. Membranes were probed with anti-NACA and anti-GAPDH antibodies (as loading controls). Specific binding to NACA proteins is denoted by the arrow and NS indicates non-specific binding of the NACA-antibody. Full-length blots are presented in Supplementary Figure S4. (**F**) RT-qPCR analysis of mRNA expression of *Usp53* in stably transfected MC3T3-E1 cells (shScr, shαNAC.1, or shαNAC.2) treated with vehicle or 100 nM PTH(1–34) for 2 h. Data are expressed relative to levels measured in the shScr expressing cells treated with vehicle. Results are means ± SD of three independent experiments (n = 3). ^#^*P* < 0.0001; ANOVA with Bonferroni’s *post-hoc* test for panels **B**, **D**, and **E**; unpaired *t* test was used for panel **C**.

To further our understanding of the significance of the PTH-activated NACA pathway in bone, we performed anti-NACA Chromatin Immunoprecipitation with deep sequencing (ChIP-Seq) in PTH-treated MC3T3-E1 osteoblast-like cells to identify genome-wide DNA-binding sites of NACA. This strategy identified a limited number of peaks (Supplementary Fig. S1A) when compared to the ChIP-Seq performed using naive IgG suggesting that PTH-activated NACA transcriptional targets are not abundant (Gene Expression Omnibus Accession number: GSE147070). These peaks mapped close to the transcription start site (TSS) within the proximal promoter region and included a match to the NACA binding consensus sequence 5′-g/cCAg/cA-3′ (Supplementary Fig. S1A). One candidate gene target for NACA in osteoblasts identified by this strategy was Ubiquitin-Specific Peptidase 53 (*Usp53*) (Fig. 1A). Binding of NACA to the proximal promoter of *Usp53* was confirmed by ChIP-PCR on MC3T3-E1 cells stimulated with PTH (1–34) or vehicle under the same conditions (Fig. 1B). We amplified the *Usp53* promoter region corresponding to the peak identified by ChIP-Seq. Using an anti-phosphoS99-NACA-specific antibody, results show a significant enrichment of the *Usp53* promoter by NACA in vehicle-treated cells as compared to naive IgG. This enrichment was further enhanced by fourfold following 100 nM PTH (1–34) treatment for 30 min. Similar results were obtained using a pan-specific anti-NACA antibody (Fig. 1B). In parallel, PTH-responsive genes were identified by RNA-Seq gene expression profiling in PTH-treated MC3T3-E1 cells. The RNA-Seq analysis identified a total of 37,479 gene targets, of which the expression of 34,623 were unchanged while 2856 were either significantly up (695) or down-regulated (1392) by twofold or more (95% confidence; moderated t-test) (Supplementary Fig. S1B). GO term analysis of regulated gene targets revealed multiple functional groups associated with skeletal biology. The top GO terms from top 3 annotation clusters for upregulated genes from DAVID are: positive regulation of transcription (*P* = 3.6E−12), glucocorticoid receptor binding (*P* = 7.5E−5), and MAP Kinase tyrosine/serine/threonine phosphatase activity (*P* = 1.3E−5) (Supplementary Fig. S1B). Moreover, the functional analysis of down-regulated genes uncovered candidates associated with transcription and DNA binding (*P* = 9.1E−20), cell cycle (*P* = 2.5E−12), and chromosome segregation (*P* = 3.0E−7) (Supplementary Fig. S1B). A further evaluation of regulated candidate genes highlighted the enrichment of signaling pathways associated with major cellular skeletal functions (Supplementary Fig. S1C). PTH(1–34) treatment upregulated genes that are closely-related to osteoblast and osteoclast differentiation and downregulated genes associated with cell cycle and cellular survival (Supplementary Fig. S1C).

RNA-Seq performed on MC3T3-E1 cells stimulated for 60 min with 100 nM PTH (1–34) showed a threefold increase in *Usp53* transcript levels as compared to vehicle-treated cells (Fig. 1C). This increase in *Usp53* transcript levels was further confirmed using quantitative RT-qPCR in primary calvarial osteoblasts treated with vehicle or 100 nM PTH (1–34) at different time points. The expression of *Usp53* increased after 1 h of treatment, peaked at 2 h of treatment and then returned to basal levels by 6 h (Fig. 1D).

The ChIP-Seq data indicated the binding of NACA to the proximal region of the *Usp53* promoter. In order to investigate the role of NACA in *Usp53* gene regulation, we used a short hairpin RNAs (shRNAs)-mediated knockdown approach to target *Naca* in MC3T3-E1 cells. The efficiency of NACA knockdown (using shNACA.1 or shNACA.2) was demonstrated by immunoblotting compared to MC3T3-E1 cells stably expressing a scrambled shRNA (shScr) (Fig. 1E). We then checked the effect of NACA knockdown on *Usp53* mRNA expression levels. The knockdown of NACA did not impact the expression levels of *Usp53* at the basal levels (Fig. 1F). PTH(1–34) stimulation of shScr-expressing MC3T3-E1 cells increased the expression levels of *Usp53* by threefold. The PTH(1–34) response was completely blunted in NACA knockdown MC3T3-E1 cells (Fig. 1F).

Together, our data show that *Usp53* is a PTH target in osteoblasts, where NACA can be involved directly or indirectly in the regulation of its expression following PTH treatment. The data also indicate that NACA contributes to the PTH-mediated transcriptional induction of *Usp53*.

### *Usp53* promoter hosts a functional binding site for NACA

**Figure 2:**
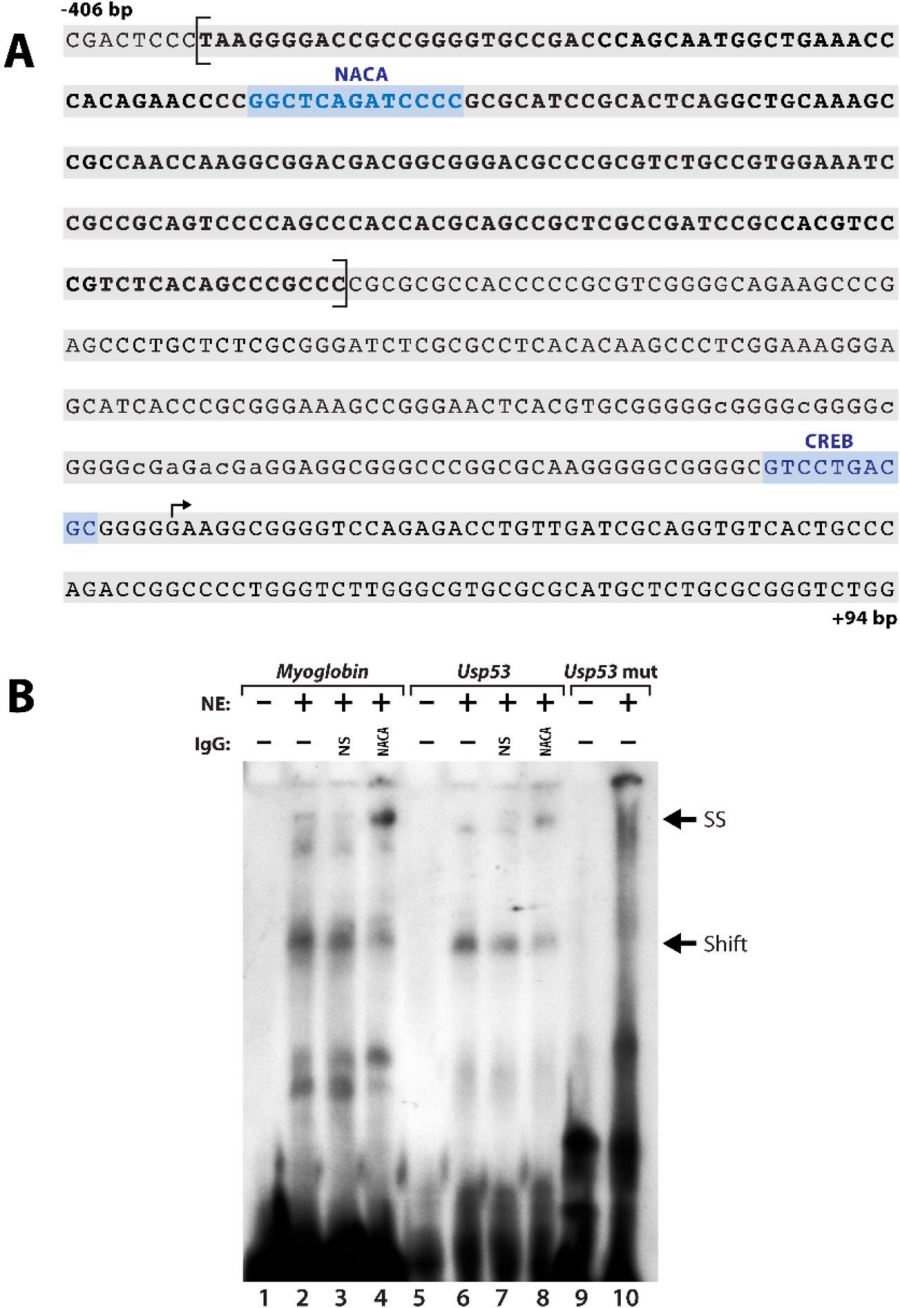
The proximal promoter of *Usp53* contains binding sites for NACA and CREB. (**A**) Mouse *Usp53* promoter sequence from nucleotide − 406 to nucleotide + 94 relative to the transcriptional initiation site (arrow). Nucleotides corresponding to the peak identified by ChIP-Seq are bracketed and in bold. Putative binding sites for NACA and CREB (highlighted) were called using Mat Inspector (Genomatix software suite). (**B**) Electrophoretic mobility shift assay (EMSA) analysis showing the binding of NACA to its binding site in the *Usp53* promoter. EMSA was performed using probes harboring the NACAbds sequence present in the *Myoglobin* (lanes 1–4) or the *Usp53* promoter (lanes 5–8), or a probe with a mutated NACAbds sequence present in the *Usp53* promoter (*Usp53* mut; lanes 9 and 10). Nuclear extracts (NE) proteins from HEK293 cells overexpressing NACA and treated with 6Bnz-cAMP to induce nuclear translocation were incubated with the probes. Negative binding was assessed by incubating the probes with the binding buffer (lanes 1 and 5). The black arrow (Shift; bottom arrow) indicates the generation of a slower migrating EMSA product, the resultant of NACA binding to NACAbds sequences in both the *Myoglobin* and *Usp53* probes. Addition of anti-NACA antibody led to an additional supershift complex (SS; top arrow), not detected when adding naive non-specific (NS) IgG to the binding reactions.

Our data show that NACA has an impact on the transcriptional regulation of *Usp53* following PTH(1–34) stimulation. We have previously characterized the DNA binding domain of the NACA protein to a loose consensus sequence centered around 5′-g/cCAg/cA-3′^20^. In silico analysis of the *Usp53* promoter region between nucleotides − 1000 and + 94 using MatInspector software^21^ identified one potential binding site for NACA and one binding site for the cAMP response element binding protein (CREB) (Fig. 2A). The predicted proximal binding site of NACA to the promoter (5′-GGCTCAGATCCCC-3′, between nucleotides − 333 and − 345) lies within the peak identified by ChIP-Seq analysis (Fig. 2A).

In order to further validate the binding of NACA to its potential binding site (bds) within the *Usp53* promoter, we performed an electrophoretic mobility shift assay (EMSA) using probes corresponding to NACAbds. The binding of NACA to the *Myoglobin* promoter was used as a positive control in these experiments^20^. The formation of mobility shift complexes (Fig. 2B, lanes 2 and 6) confirmed the binding of proteins from NACA-enriched nuclear extracts to the probes harboring NACAbds. We further confirmed the presence of NACA in the shifted DNA–protein complexes. The addition of anti-NACA antibody to the reactions super shifted (SS) the formed complexes (Fig. 2B, lanes 4 and 8). This supershift was not observed when adding naïve IgG (Fig. 2B, lanes 3 and 7). In order to further validate the fidelity of NACA binding, we mutated its potential binding site within the *Usp53* promoter. The 5′-GGCTCAGATCCCC-3′ NACA binding site was mutated to 5′-GGCTCgGgTCCCC-3′. The formation of a DNA–protein complex was abrogated when incubating NACA protein extracts with mutated NACAbds probes (Fig. 2B, lane 10). These experiments confirm the binding of NACA to its cognate site within the *Usp53* proximal promoter.

### NACA is implicated in the regulation of the *Usp53* promoter

**Figure 3:**
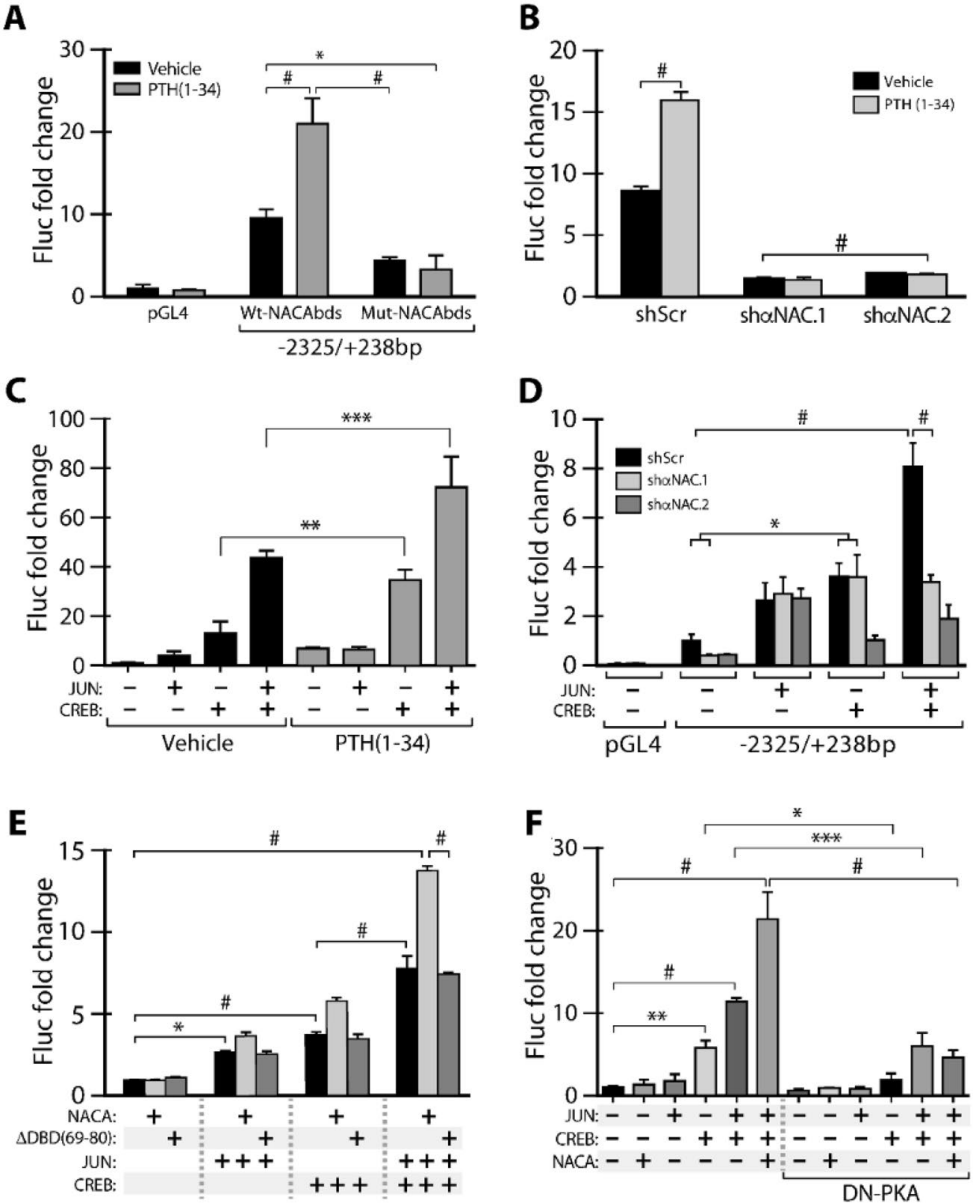
NACA, JUN, and CREB are implicated in *Usp53* promoter regulation. (**A**) Firefly luciferase (Fluc) reporter assay was performed using MC3T3-E1 cells overexpressing wildtype or mutated *Usp53*-Luc vector (-2325/+ 238 bp). The mutated region lies in the proximal NACA binding site of the *Usp53*-Luc vector. (**B**) Firefly luciferase (Fluc) reporter assay were performed using MC3T3-E1 cells stably expressing shRNAs targeting *Naca* (shαNAC.1 and shαNAC.2) or scrambled shRNA (shScr) as a control by lentiviral infection. When indicated, cells were treated with vehicle or 100 nM PTH (1–34) for 24 h before performing the assay. (**C**) Firefly luciferase (Fluc) reporter assay in MC3T3-E1 cells transfected with *Usp53*-Luc vector (− 2325/+ 238 bp) alone or along with JUN and/or CREB expression vectors treated with vehicle or 100 nM PTH(1–34) for 24 h. (**D**) Firefly luciferase (Fluc) reporter assay in MC3T3-E1 cells stably expressing shRNAs against *Naca* (shNACA.1 and shNACA.2) or scrambled shRNA (shScr), transfected with *Usp53*-Luc vector (-2325/+ 238 bp) alone or along with JUN and/or CREB expression vectors. Cells were treated with 100 nM PTH (1–34) for 24 h before performing the assay. (**E**) Firefly luciferase (Fluc) reporter assay in MC3T3-E1 cells transfected with *Usp53*-Luc vector (-2325/+ 238 bp) alone or along with NACA-S99D or NACA mutant deltaDBD (69–80), JUN and/or CREB expression vectors. Cells were treated with 100 nM PTH (1–34) for 24 h before performing the assay. (**F**) Firefly luciferase (Fluc) reporter assay in MC3T3-E1 cells transfected with *Usp53*-Luc vector (-2325/+ 238 bp) alone or along with 50 ng of NACA, JUN and/or CREB expression vectors, with or without 100 ng of dominant negative DN-PKA expression vector. Cells were treated with 100 nM PTH(1–34) for 24 h before performing the assay. Empty pGL4 vector was used as a negative control. Firefly luciferase counts were normalized to Renilla counts. Results are presented as the mean fold induction ± SD. *P ≤ 0.05; **P ≤ 0.01; ***P ≤ 0.001; ^#^*P* < 0.0001; ANOVA with Bonferroni’s *post-hoc* test for all panels.

We cloned a fragment of the *Usp53* promoter corresponding to nucleotides − 2325 to + 238 bp (− 2325/+ 238 bp) relative to the annotated transcription start site into the pGL4.10[LUC2] reporter vector. In order to check the functionality of the NACA binding site, we implemented site-directed mutagenesis and shRNA-mediated knockdown techniques. The 5′-GGCTCAGATCCCC-3′ NACA binding site was mutated to 5′-GGCTCgGgTCCCC-3′ in the context of the − 2325/+ 238 bp promoter fragment. The cloned promoter fragment (− 2325/+ 238 bp) carrying a wild-type response element for NACA (Wt-NACAbds) showed a tenfold increase in the transcriptional activity of the luciferase reporter as compared to cells transfected with pGL4 vector (Fig. 3A). The *Usp53* promoter activity was reduced by 40% when we transfected the same fragment carrying a mutated sequence of the NACA response element (Mut-NACAbds) (Fig. 3A, *black bars*). The mutation abrogated the PTH-induced activation of the cloned *Usp53* fragment, resulting in > 50% reduction in the promoter activity (Fig. 3A, *grey bars*). Interestingly, the knockdown of NACA using shαNAC.1 or shαNAC.2 shRNAs resulted in > 80% reduction in *Usp53* promoter activity as compared to reporter gene expression of MC3T3-E1 cells transfected with shScr (Fig.3B, *black bars*). This impact on basal levels of *Usp53* expression from the cloned promoter (Fig. 3B, *black bars*) as compared to what was observed on basal level expression of the endogenous gene (Fig. 1E) most probably reflects differences between naked plasmid DNA vs. endogenous gene in its native chromatin environment. The absence of NACA blunted the induction of the *Usp53* promoter fragment by PTH (Fig.3B, *grey bars*). Taken together, these results show that the presence of NACA and its proper binding to its response element is critical for promoter activity.

### NACA is required for the JUN/CREB-mediated synergistic regulation of the *Usp53* promoter

A potential binding site for CREB lies between nucleotides − 5 and − 14 relative to the annotated transcription initiation site, within the *Usp53* proximal promoter (Fig. 2A). The in silico predictions suggest a potential role for CREB along with NACA in the transcriptional regulation of the promoter. We have recently observed that both NACA and CREB can regulate the transcription of the *Nfil3* promoter in MC3T3-E1 cells^19^. Besides, previous studies have highlighted the need for a *bona fide* transcription factor partner such as the basic domain-leucine zipper (bZIP) homodimeric AP-1 family member JUN to regulate transcription, as NACA lacks a transcriptional activation domain^17,22^. Considering this, we tested whether CREB along with JUN activate the transcription of the cloned promoter fragment alone or in combination with NACA.

We first assessed the JUN-CREB regulation of the *Usp53* cloned fragment at basal levels and in response to PTH(1–34) stimulation in MC3T3-E1 cells. At basal levels, the overexpression of 50 ng of JUN expression vector did not induce a statistically significant increase in the promoter activity. However, the transcriptional activity of the cloned promoter fragment (-2325/+ 238 bp) was significantly enhanced following the overexpression of 50 ng of CREB and further potentiated when co-expressing CREB and JUN together (Fig. 3C, *black bars*). The CREB-mediated and the dual activation by JUN and CREB were dramatically increased following PTH(1–34) treatment for 24 h (Fig. 3C, *grey bars*). This data highlighted the combinatorial actions of the JUN/CREB complex in regulating the *Usp53* promoter activity at basal levels and following PTH(1–34) treatments.

We then assessed the JUN-CREB activation of the *Usp53* cloned fragment in *Naca*-knockdown cells. The transcriptional activity of the cloned promoter fragment (-2325/+ 238 bp) was higher than the empty pGL4 vector (Fig. 3D, *black bars*). As previously demonstrated in Fig. 3C, similar trends of promoter activation were obtained following CREB overexpression or co-expression of CREB and JUN together in MC3T3-E1 cells transfected with shScr (Fig. 3D, *black bars*). Interestingly, this additive activation was completely abolished in MC3T3-E1 cells transfected with NACA shRNAs (Fig. 3D, *light and dark grey bars*). It is important to note that the absence of NACA did not affect the promoter activation when either JUN or CREB were transfected alone (Fig. 3D, *light and dark grey bars*).

We then investigated whether the binding of NACA to DNA is needed for the optimal promoter activation carried by JUN and CREB. To mimic the PTH(1–34) effect on NACA translocation, we overexpressed NACA-S99D mutant; a phosphomimetic form of NACA that shows enhanced nuclear localization^14^. As described in Fig. 3D, similar patterns of promoter activation were seen when overexpressing JUN and CREB, alone or combined (Fig. 3E, *black bars*). The overexpression of NACA-S99D mutant or a NACA mutant lacking its DNA binding domain (delta DBD(69–80)) had no effect on the JUN or CREB-mediated activation of the promoter fragment (Fig. 3E, *light and dark grey bars*). However, overexpressing NACA-S99D along with JUN and CREB in MC3T3-E1 cells boosted the promoter activity as compared to conditions with JUN and CREB alone (Fig. 3E, *grey bar*). This enhanced activity was not seen when overexpressing the delta DBD (69–80) NACA mutant in the same condition (Fig. 3E, *dark grey bar*).

We interpret this data to mean that NACA binding to its cognate site is important for the combined actions of JUN and CREB in the regulation of *Usp53* promoter activity.

### The NACA/JUN/CREB activation of the *Usp53* promoter is mediated by PKA

Our data (Fig. 3C–E) have demonstrated the PTH-induced CREB activation of the *Usp53* promoter. One interesting aspect of this regulatory mechanism is the involvement of protein kinase A (PKA) in this activation. To test this possibility, we have blocked endogenous PKA signaling by expressing a dominant-negative form of PKA (DN-PKA)^23^ and assessed the integrity of the NACA/JUN/CREB activation. The transcriptional activation of the *Usp53* promoter by CREB alone, CREB/JUN together or NACA/JUN/CREB combined was completely abolished following the overexpression of 100 ng of DN-PKA expression vector (Fig. 3F). This data indicates that PKA is implicated in the regulation of the *Usp53* promoter by the NACA/JUN/CREB trio in MC3T3-E1 cells.

### *Usp53* affects mesenchymal cell lineage selection and differentiation in vitro

Two main isoforms of *Usp53* have previously been described: a full-length isoform comprising 15 coding exons and a short isoform containing exons 1–7 of the Usp53 gene^6^. Little is known about the expression of *Usp53* in different lineages. We analyzed the expression of the two isoforms by RT-qPCR in long and flat bones as well as in fat compartments of wild-type mice at 3-weeks of age. *Usp53* was expressed in calvaria and tibia and in white (gonadal) and brown (interscapular) fat. In all tissues tested, the expression of the long, full-length isoform was predominant (Supplementary Fig. S2).

**Figure 4:**
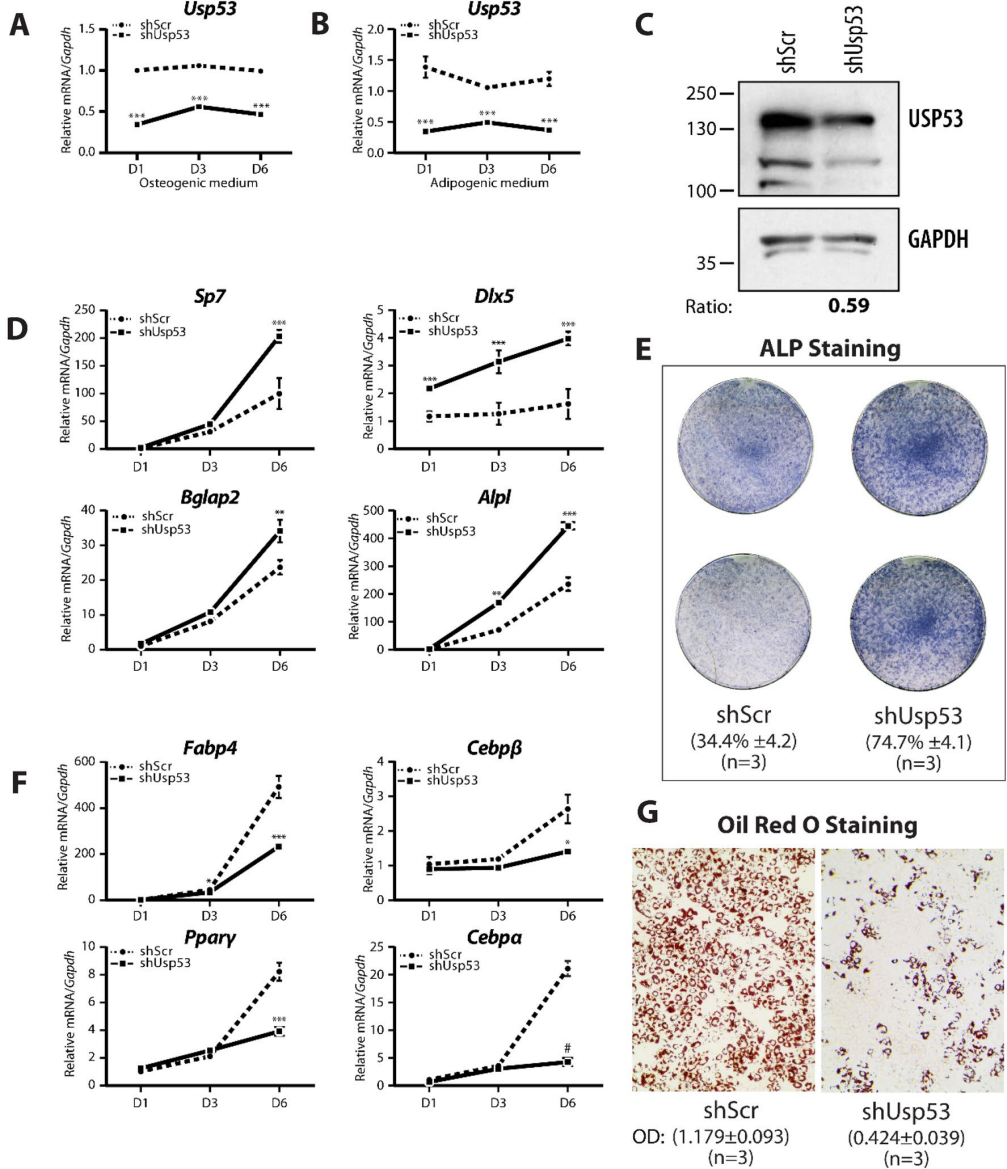
*Usp53* controls osteogenic and adipogenic fate determination in vitro*.* ST2 cells expressing scrambled shRNAs (shScr) targeting control and shRNAs targeting *Usp53* were grown under osteogenic conditions (**A**) or adipogenic conditions (**B**) for 6 days. (**C**) Immunoblot of whole cells extracts from ST2 cells stably expressing shRNA targeting *Usp53* (shUsp53) and scrambled shRNA (shScr) as control. Membranes were probed with anti-USP53 and anti-GAPDH antibodies (as loading controls). Relative signal intensity quantified using ImageJ is shown below each lane. Full-length blots are presented in Supplementary Figure S5. Gene expression analysis of osteogenic (**D**) or adipogenic (**F**) differentiation markers. Quantitative RT-qPCR using TaqMan probes against osteoblast or adipocyte differentiation markers, normalized to *Gapdh*, was performed on RNA isolated from shScr-stably transfected (dashed line) or shUsp53-stably transfected (solid line) pools of ST2 cells grown for 6 days in osteogenic or adipogenic medium. ST2 cultures were examined for ALP staining at day 12 (**E**) or oil red O at day 6 (**G**). Results are presented as the mean fold change $\pm$ SD. *P ≤ 0.05; **P ≤ 0.01; ***P ≤ 0.001; ^#^*P* < 0.0001; ANOVA with Bonferroni’s *post-hoc* test for all panels.

**Figure 5:**
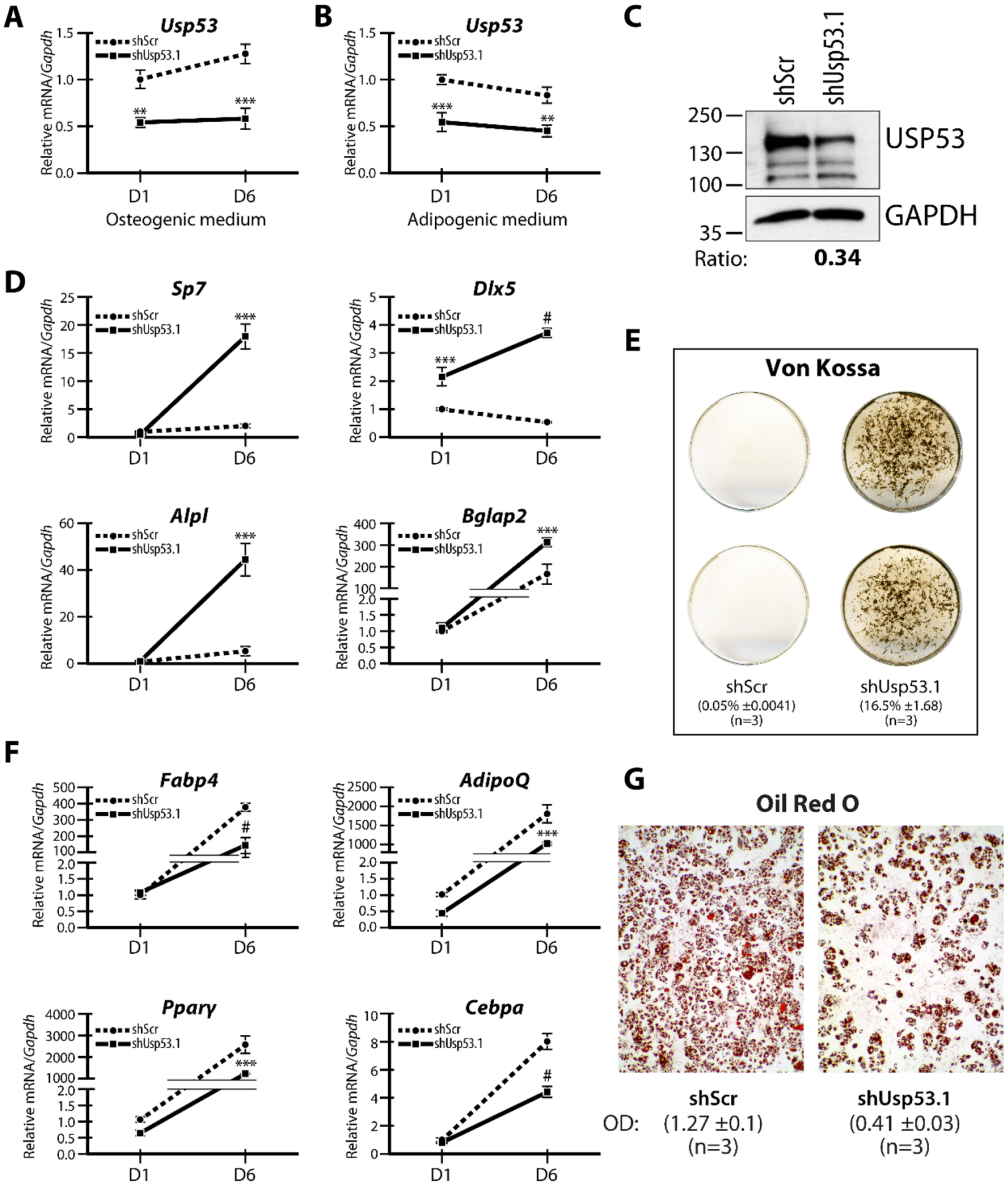
*Usp53* knockdown with a different shRNA confirms the impact on osteogenic and adipogenic fate determination in vitro*.* ST2 cells expressing scrambled shRNAs (shScr) targeting control and shRNAs targeting *Usp53* were grown under osteogenic conditions (**A**) or adipogenic conditions (**B**) for 6 days. (**C**) Immunoblot of whole cells extracts from ST2 cells stably expressing shRNA targeting *Usp53* (shUsp53.1) and scrambled shRNA (shScr) as control. Membranes were probed with anti-USP53 and anti-GAPDH antibodies (as loading controls). Relative signal intensity quantified using ImageJ is shown below each lane. Full-length blots are presented in Supplementary Figure S6. Gene expression analysis of osteogenic (**D**) or adipogenic (**F**) differentiation markers. Quantitative RT-qPCR using TaqMan probes against osteoblast or adipocyte differentiation markers, normalized to *Gapdh*, was performed on RNA isolated from shScr-stably transfected (dashed line) or shUsp53-stably transfected (solid line) pools of ST2 cells grown for 6 days in osteogenic or adipogenic medium. ST2 cultures were examined for von Kossa staining at day 8 (**E**) or oil red O at day 6 (**G**). Results are presented as the mean fold change ± SD. **P ≤ 0.01; ***P ≤ 0.001; ^#^*P* < 0.0001; ANOVA with Bonferroni’s *post-hoc* test for all panels.

**Figure 6:**
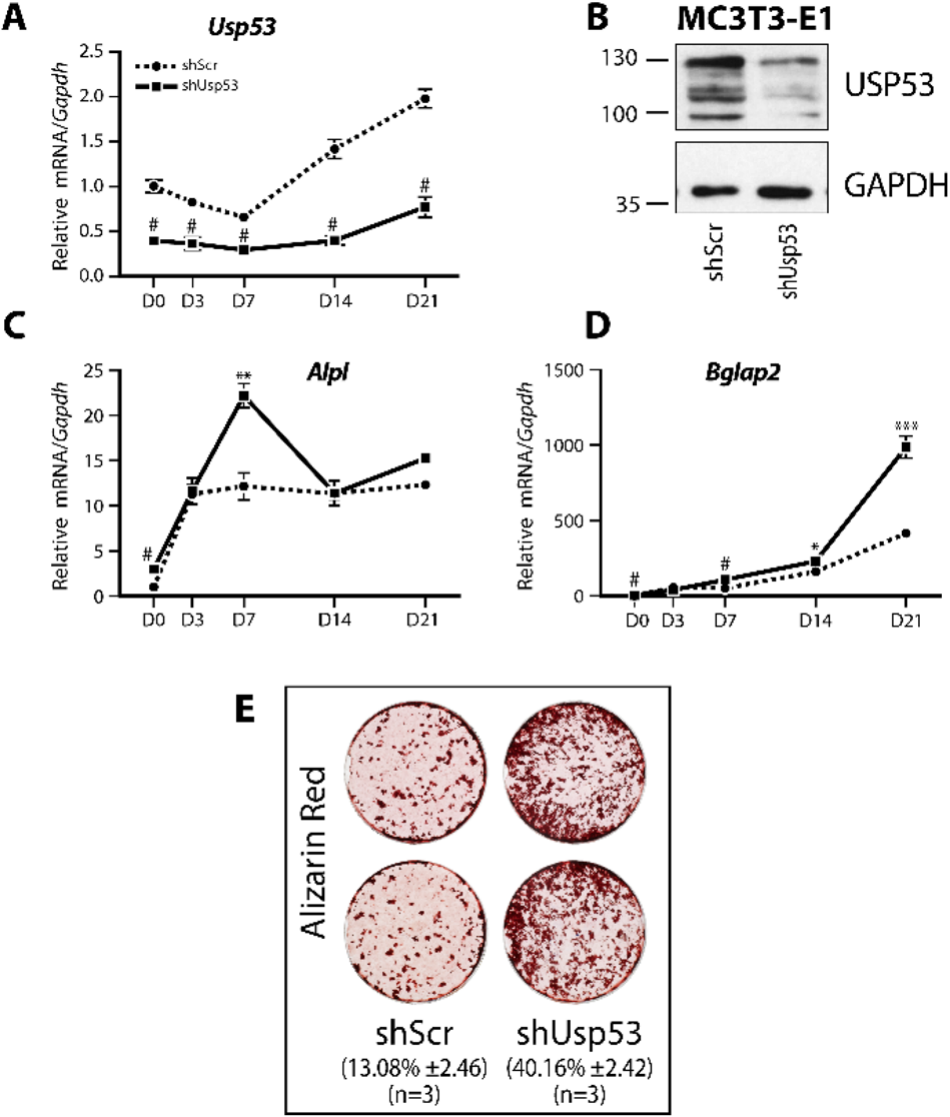
*Usp53* knockdown enhances the differentiation of committed osteoblasts. (**A**, **C**, **D**) Knockdown of *Usp53* in MC3T3-E1 cells and gene expression analysis of osteogenic differentiation markers. Quantitative RT-qPCR using probes against *Usp53*, *Alpl*, and *Bglap2*, normalized to *Gapdh*, was performed on RNA isolated from shScr-stably transfected (dashed line) or shUsp53-stably transfected (solid line) pools of MC3T3-E1 cells differentiated for 21 days in osteogenic medium. (**B**) Western blot analysis of Usp53 expression. Total protein isolated from MC3T3-E1 cells stably transfected with shScr or shUsp53 at D1, confirming the knockdown of *Usp53*. GAPDH was used as a loading control. Full-length blots are presented in Supplementary Figure S7. (**E**) MC3T3-E1 cultures were examined for calcium deposition using alizarin red staining at D21. Results are presented as the mean fold change ± SD. *P ≤ 0.05; **P ≤ 0.01; ***P ≤ 0.001; ^#^*P* < 0.0001; ANOVA with Bonferroni’s *post-hoc* test for all panels.

To examine the putative role of *Usp53* in mesenchymal cell differentiation, we targeted its expression in ST2 stromal cells. Short hairpin RNAs (shRNAs) targeting *Usp53* (shUsp53) or a control scrambled shRNA (shScr) were transfected into ST2 cells and stable pools expressing the distinct shRNAs were established. The stably transfected cells were differentiated in either osteogenic or adipogenic medium for 6 days. The expression levels of *Usp53* showed a significant 50% reduction following knockdown and remained stable through the osteoblast differentiation regimen (Figs. 4A, 5A and 6A). Knockdown of *Usp53* in ST2 and MC3T3-E1 cells using two different shUsp53 vectors resulted in significant reduction in USP53 protein levels (Figs. 4C, 5C and 6B).

We then monitored the level of transcripts expressed during early- and late-stage osteoblastic differentiation. The knockdown of *Usp53* enhanced the expression of early-stage osteoblast markers, such as *Osterix* (*Sp7*) and *Dlx5* as well as late-stage osteoblast markers such as osteocalcin (*Bglap2*) and alkaline phosphatase (*Alpl*) (Fig. 4D). Alkaline phosphatase activity was markedly increased in the absence of *Usp53* (from 34.4 ± 4.2% in controls to 74.7 ± 4.1% in *Usp53* knockdown cells; *P* = 0.0001; *t*-test), assessed by staining at Day 6 (Fig. 4E). These results show that the absence of *Usp53* enhanced osteoblast differentiation. This data was further validated using another shRNA against *Usp53* to eliminate the probability of any off-target effects associated with shRNA use (Fig. 5A, C–E).

Similar results were obtained upon analysis of MC3T3-E1 osteoblast-like cells. *Usp53*-knockdown MC3T3-E1 cells exhibited a drastic increase in osteocalcin (*Bglap2*) and *Alpl* expression levels throughout differentiation (Fig. 6C, D) together with increased calcium deposition (from 13.1 ± 2.5% in controls to 40.2 ± 2.4% in *Usp53* knockdown cells; *P* = 0.0001; *t*-test) as assessed by Alizarin Red Staining (Fig. 6E). We interpret these results to mean that *Usp53* inhibits osteoblastogenesis of both bipotential stromal cells and committed osteoblasts.

We also cultured the *Usp53* knockdown ST2 cells in adipogenic medium for 6 days. Again, the expression levels of *Usp53* were stable throughout adipocyte differentiation and significant knockdown was measured (Fig. 4B). Interestingly, *Usp53*-deficient ST2 cells cultured in adipogenic media showed decreased induction of adipogenic activators such as *Fabp4*, *Cebpα/β*, and *Pparγ* (Fig. 4F). This translated into decreased lipid accumulation (optical density of staining decreased from 1.2 ± 0.1% in shScr-transfected control cells to 0.4 ± 0.04% in *Usp53* knockdown cells; *P* < 0.0001; *t*-test) as assessed by Oil Red O Staining at Day 6 (Fig. 4G). Thus, the absence of *Usp53* caused a substantial reduction in adipocytic differentiation capacity. Again, these observations were validated using another shRNA against *Usp53* to avoid off-target effects associated with shRNA use (Fig. 5B, F, G).

Taken together, our data suggest that *Usp53* is a regulator of the lineage-making decisions of mesenchymal cells, altering their differentiation into osteoblasts or adipocytes reciprocally.

### PTH responses in *Usp53* knockdown osteoblasts and adipocytes

**Figure 7:**
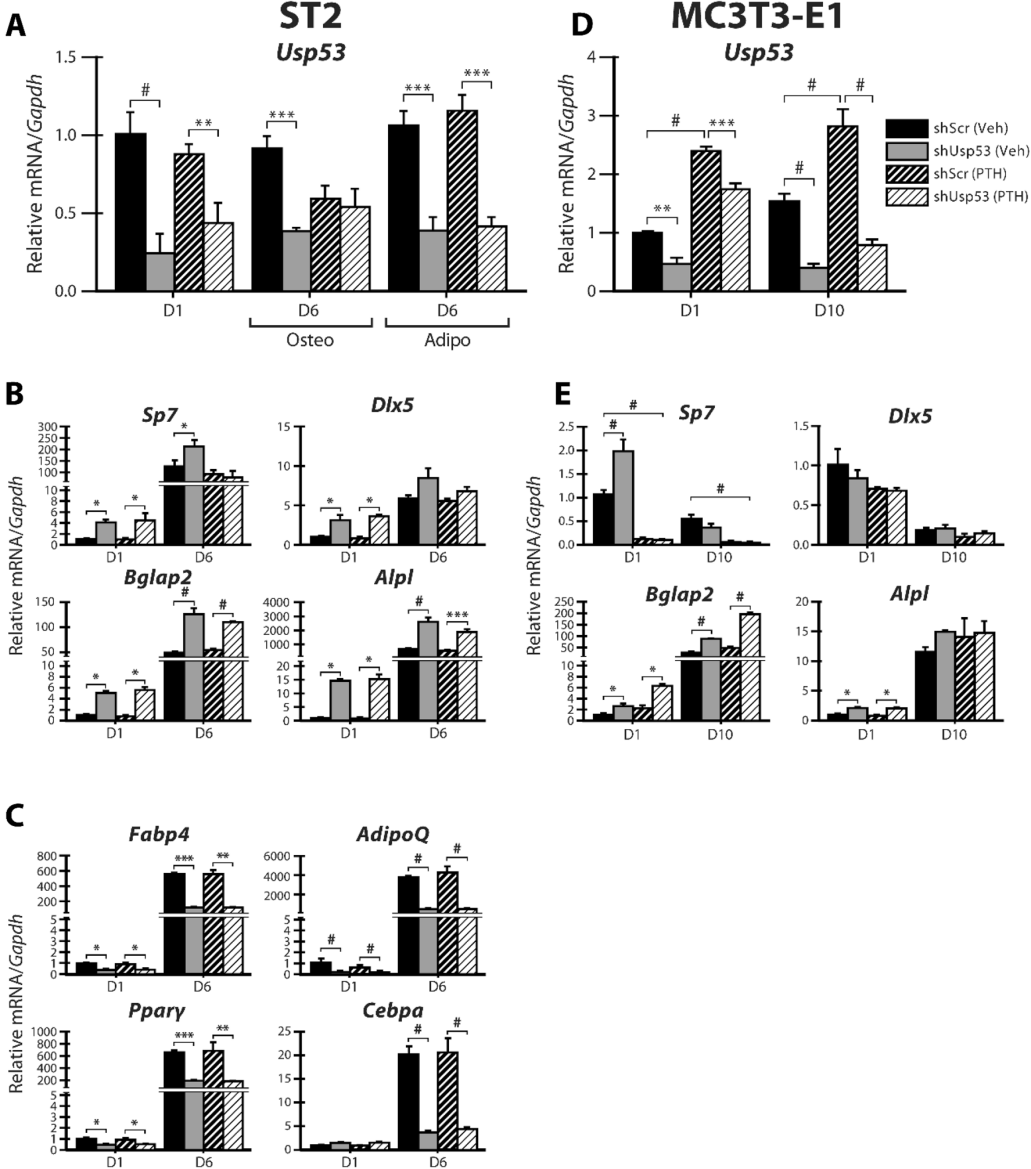
PTH responses in *Usp53* knockdown osteoblasts and adipocytes. ST2 cells expressing scrambled shRNAs (shScr) targeting control and shRNAs targeting *Usp53* were grown under osteogenic or adipogenic conditions for 6 days. Gene expression analysis of *Usp53* (**A**) osteogenic (**B**) or adipogenic (**C**) differentiation markers. Quantitative RT-qPCR using TaqMan probes against osteoblast or adipocyte differentiation markers, normalized to *Gapdh*, was performed on RNA isolated from shScr-stably transfected or shUsp53-stably transfected pools of ST2 cells grown for 6 days in osteogenic or adipogenic medium treated with vehicle or 100 nM PTH(1–34) for 2 h prior to analysis. Gene expression analysis of *Usp53* (**D**) or osteogenic (**E**) differentiation markers of MC3T3-E1 cells. Quantitative RT-qPCR using TaqMan probes against osteoblast differentiation markers, normalized to *Gapdh*, was performed on RNA isolated from shScr-stably transfected or shUsp53-stably transfected pools of MC3T3-E1 cells grown for 10 days in osteogenic medium treated with vehicle or 100 nM PTH(1–34) for 2 h prior to analysis. Results are presented as the mean fold change ± SD. *P ≤ 0.05; **P ≤ 0.01; ***P ≤ 0.001; ^#^*P* < 0.0001; ANOVA with Bonferroni’s *post-hoc* test for all panels.

We have shown that *Usp53* expression is induced following PTH(1–34) treatment of MC3T3-E1 cells. To determine whether *Usp53* alters PTH responses in cultured cells, we treated *Usp53*-knockdown ST2 and MC3T3-E1 cultures with PTH(1–34) at different timepoints through differentiation. The expression levels of *Usp53* showed a significant 70% reduction following knockdown and remained stable through the osteoblast (osteo) and adipocyte (adipo) differentiation regimens (Fig. 7A, *solid grey bars*). PTH(1–34) stimulation for 2 h did not induce any significant changes in *Usp53* expression levels of shScr (Fig. 7A, *dashed black bars*) or shUsp53-stably expressing ST2 cells (Fig. 7A, *dashed grey bars*), cultured in osteogenic or adipogenic medium for 6 days (Fig. 7A).

Looking into the osteogenic differentiation of *Usp53*-knockdown ST2 cultures, we assessed the changes in early- and late-stage osteogenic differentiation markers at basal levels and following PTH(1–34) stimulation. At D1 of differentiation, the knockdown of *Usp53* enhanced the expression of *Sp7*, *Dlx5*, *Bglap2*, and *Alpl* significantly in both vehicle- (Fig. 7B, *solid grey bars*) and PTH(1–34)-treated (Fig. 7B, *dashed grey bars*) cultures. At D6 of differentiation, the increase in *Sp7* expression levels in vehicle-treated cultures (Fig. 7B, *solid grey line*) was abolished following PTH(1–34) treatment for 2 h (Fig. 7B, *dashed grey line*). On the other hand, the knockdown of *Usp53* did not alter the expression of *Dlx5* at D6 of differentiation in both vehicle-(Fig. 7B, *solid grey bar*) and PTH(1–34)-treated (Fig. 7B, *dashed grey bar*) cultures. Moreover, the enhanced expression of *Bglap2* and *Alpl* in *Usp53*-knockdown cultures at basal levels (Fig. 7B, *solid grey bars*) was unchanged following PTH(1–34) treatment (Fig. 7B, *dashed grey bars*) at D6 of differentiation.

Assessing the adipogenic differentiation of *Usp53*-knockdown ST2 cultures, we found that loss of *Usp53* inhibits the expression of *Fabp4*, *AdipoQ*, and *Pparg* in Vehicle-treated ST2 cultures through differentiation (Fig. 7C, *solid bars*). The decreased expression of adipogenic markers was not altered by PTH(1–34) stimulation (Fig. 7C, dashed bars). *Cebpα* was exclusively downregulated at D6 of adipogenic differentiation in *Usp53*-knockdown ST2 cultures (Fig. 7C, *solid grey bar*) and PTH(1–34) treatment of those cultures had no effect on the decreased expression of the gene (Fig. 7C, *dashed grey bar*).

The same approach was used to characterize the impact of *Usp53* knockdown on PTH responses in committed osteoblast cells. The expression levels of *Usp53* showed a significant 60% reduction following knockdown and remained stable through MC3T3-E1 differentiation regimen (Fig. 7D, *solid grey bars*). PTH(1–34) stimulation for 2 h increased the expression levels of *Usp53* at D1 and D10 of osteoblastic differentiation, but the levels remained significantly reduced in knockdown cells (Fig. 7D, *dashed bars*). When changes in the expression levels of osteoblast differentiation markers were monitored, *Usp53*-knockdown MC3T3-E1 cultures exhibited increased expression levels of *Sp7* in vehicle-treated (Fig. 7E, *solid grey bars*) cultures at D1. PTH(1–34) treatment for 2 h blocked the expression of *Sp7* to a similar magnitude in both shScr- and shUsp53-stably expressing MC3T3-E1 cultures at D1 and D10 (Fig. 7E, *dashed black and grey bars*). Moreover, the knockdown of *Usp53* increased the expression levels of *Bglap2* throughout differentiation (Fig. 7E, *solid bars*) and PTH(1–34) treatment had no impact on this increase (Fig. 7E, *dashed bars*). Expression levels of *Alpl* were enhanced by *Usp53*-knockdown (Fig. 7E, *solid bars*) exclusively at D1, with no significant modulation post PTH(1–34) stimulation (Fig. 7E, *dashed bars*). Finally, the knockdown of *Usp53* had no impact on *Dlx5* expression levels through differentiation under any condition (Fig. 7E, *solid and dashed bars*).

Our data suggest that *Usp53* expression levels impact PTH responses differentially across the osteoblastic differentiation sequence. It also indicates that the knockdown of *Usp53* does not affect PTH responses during adipogenic differentiation.

### *Usp53* affects mesenchymal cell lineage-making decisions and differentiation in vivo

**Figure 8:**
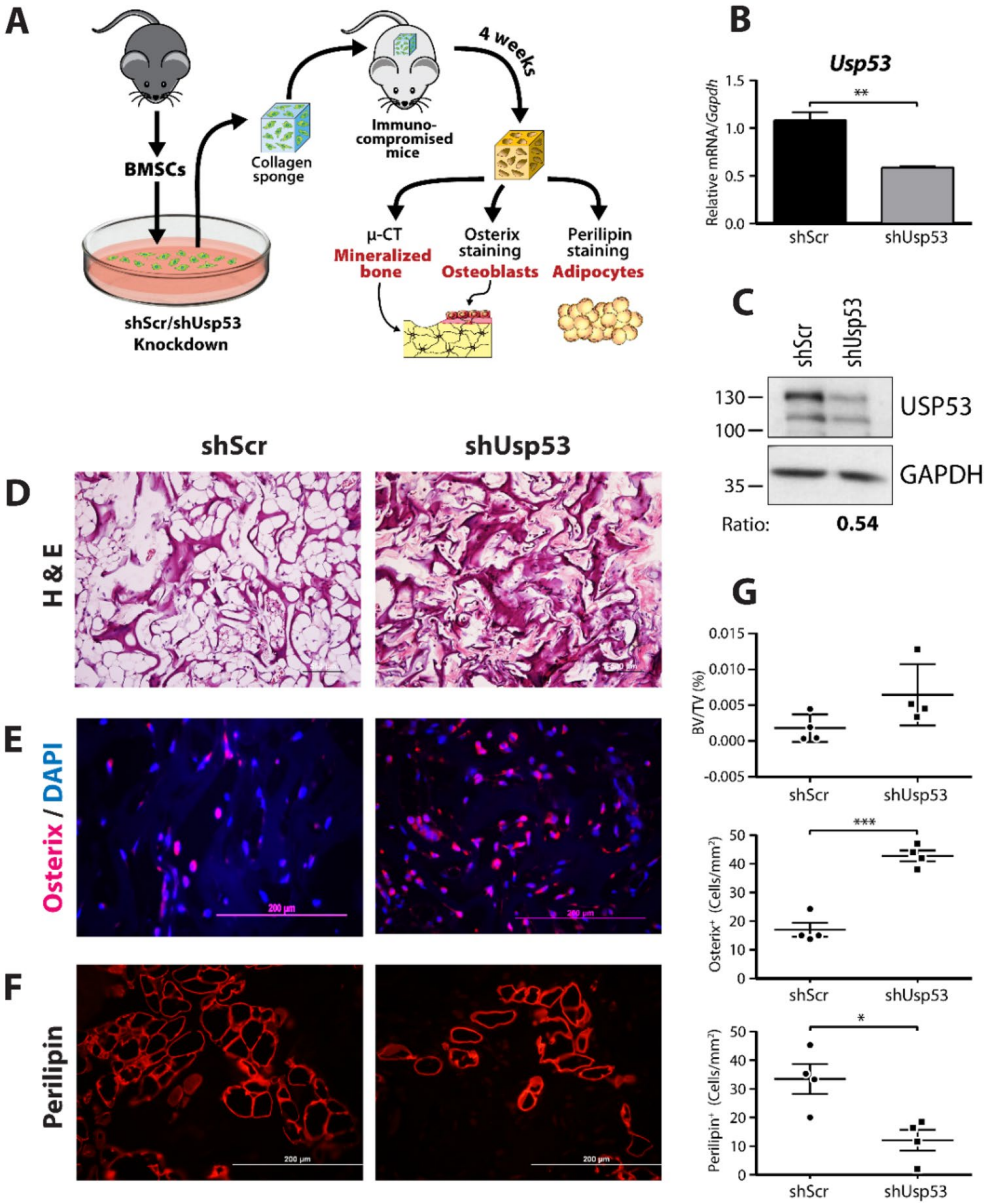
*Usp53* knockdown enhances osteoblastogenesis and inhibits adipogenesis in vivo*.* (**A**) Scheme showing the workflow of the in vivo osteogenesis assay performed on immunocompromised mice. The mouse drawing is based on a free download from flyclipart.com which was adapted for our purposes. The software used to create the diagram was Adobe Illustrator CS5 (https://www.adobe.com/ca/products/illustrator.html) (**B**) Knockdown of *Usp53* in bone marrow stromal cells (BMSCs). Quantitative RT-qPCR using TaqMan probe against *Usp53*, normalized to *Gapdh*, was performed on RNA isolated from stable BMSCs cultures transfected with shScr or shUsp53. (**C**) Immunoblot of whole cells extracts from BMSCs stably expressing shRNA targeting *Usp53* (shUsp53) and scrambled shRNA (shScr) as control. Membranes were probed with anti-USP53 and anti-GAPDH antibodies (as loading controls). Relative signal intensity quantified using ImageJ is shown below. Full-length blots are presented in Supplementary Figure S8. (**D**) H & E staining of murine implants (shScr and shUsp53) after 4-weeks of BMSCs transplantation embedded in collagen sponge. (**E**) Immunostaining of the implants (shScr and shUsp53) showing Osterix staining for osteoblasts (red) and DAPI staining for nuclei (blue). (**F**) Immunostaining of the implants (shScr and shUsp53) showing Perilipin staining for adipocytes (red). (G) Quantification of the newly formed bone in the implants using Micro-CT (upper panel); quantification of Osterix-positive osteoblast cells (middle panel); quantification of perilipin-positive adipocytes (lower panel). BV/TV, bone volume/tissue volume. *P < 0.05, **P < 0.01, ***P < 0.001; *t* test was used as statistical test in all panels.

To ensure that the observed effects were not restricted to established cell lines, we performed an in vivo differentiation assay with murine bone marrow stromal cells (BMSCs)^24^. BMSCs were isolated from femoral and tibial bone of C57BL/6 wild-type male mice and infected with shUsp53 or the control shScr and their ability to form bone or fat in vivo was assessed (Fig. 8A).

The expression level of *Usp53* showed a 50% reduction following knockdown in BMSCs (Fig. 8B). The efficiency of USP53 knockdown (using shUsp53) was demonstrated by immunoblotting compared to BMSCs stably expressing scrambled shRNA (shScr) (Fig. 8C). Cells were implanted on collagen scaffolds and inserted subcutaneously into female immunocompromised mice (Fig. 8A). Four (4) weeks after surgery, the mice were sacrificed and the implants were processed for microcomputed tomography (micro-CT) and histological analysis. Initially, sections were stained with H&E, which stains the newly deposited bone matrix. BMSCs expressing shUsp53 showed increased H&E staining (Fig. 8D). Bone formation was also assessed by micro-CT. The relative bone volume versus tissue volume (BV/TV) was higher in *Usp53* knockdown implants as compared to control, but the experimental variation prevented to reach statistical significance (Fig. 8G, top panel). To further confirm the presence of bone cells, we performed immunostaining using an anti-SP7 (Osterix) antibody to identify osteoblasts (Fig. 8E) and quantified the results by counting Osterix-positive cells in different histological sections. *Usp53* knockdown implants showed a significant increase in Osterix-positive cells (Fig. 8G, middle panel). Adipocytes were detected by immunostaining with an anti-perilipin antibody, which detects surface-associated lipid droplets present in adipocytes. *Usp53* knockdown implants showed reduced perilipin staining as compared to control (Fig. 8F), and this decrease was statistically significant (Fig. 8G, lower panel). We conclude that a decrease in *Usp53* expression enhanced osteoblastogenesis and suppressed adipogenesis in vivo.

## Discussion

In addition to *Lrp6*^18^, *Bglap2*^14^, and *Nfil3*^19^, we have added *Usp53* to the list of target genes regulated by the PTH-NACA pathway in osteoblasts. This finding is significant as the literature lacks any characterization of *Usp53* regulation or function in any tissue. The identification of *Usp53* as a target of the PTH-NACA pathway led us to investigate the transcriptional regulation of *Usp53* and assess whether *Usp53* may have a function in mesenchymal cell lineage specification.

Little is known about the upstream signaling regulating *Usp53* expression. One study has reported the induction of *USP53* in human bone marrow MSCs when treated with BMP-2 and Wnt3a together^25^. This is the first report demonstrating the regulation of *Usp53* by PTH signaling. The ChIP-Seq data reported herein show that PTH-activated NACA was recruited to the proximal promoter region of *Usp53*. The same regulatory model has been previously reported for the *Bglap2*, *Lrp6,* and *Nfil3* promoters in osteoblasts. Our work highlighted their functions in fine-tuning PTH signaling (in the case of *Lrp6*), modulating osteoblast function (for *Bglap2*), and differentially regulating osteoblast vs. osteocyte differentiation (*Nfil3*)^14,18,19^. It is yet to be determined whether the PTH-induction of *Usp53* in osteoblasts or osteocytes dictates an osteoanabolic or osteocatabolic effect on bone.

Lessons learnt from different promoters regulated by the PTH-NACA pathway helped uncover the missing pieces in the *Usp53* regulatory scheme. We have previously shown that NACA engages with bZIP family transcription factors and potentiates their activity. JUN and JUND are two partners of NACA regulating the expression of the *Bglap2* and *Lrp6* promoters, respectively^16,18^. In addition, we have recently characterized the transcriptional regulation of the *Nfil3* promoter by both NACA and CREB in osteoblasts^19^. Our data herein suggest that NACA is indispensable for *Usp53* promoter activity, its response to PTH, and its full activation by the JUN-CREB complex. We have previously demonstrated that NACA does not interact with CREB at basal levels, nor following cAMP activation or PTH(1–34) induction^19^. In this respect, it is plausible to hypothesize that JUN and CREB heterodimerize^26^ and interact with NACA to achieve the optimal potentiation of the *Usp53* promoter. We hypothesize that the JUN partner of the heterodimer mediates the interaction of the dimer with NACA. One exciting aspect is the contribution of PKA to the regulatory circuit of *Usp53*. We envisage this involvement to not only induce CREB activation, but also induce the nuclear translocation and activation of NACA via phosphorylation. This scenario is highly probable, as our previous work has dissected the PTH-Gαs-mediated regulation and activation of NACA by PKA in osteoblasts^14^.

*USP53* has been reported to be expressed in skeletal muscle, hair follicles, and cardiac muscle^27^. We have further detected its expression in bone tissue (calvaria and tibia) and adipose tissue (white fat and brown fat). The prevalence of the full-length isoform is most likely related to its function in those tissues.

Little is known about the biological function of USP53. Sequence comparisons with other members of the *USP* gene family revealed that USP53 lacks a functionally essential histidine residue within the His-box involved in the recognition of the C terminus of ubiquitin^28^, which has led authors to suggest that the protein is devoid of enzymatic activity^5,6^. The lack of proteolysis activity suggests that USP53 may function as a protein scaffold supporting the assembly of complexes implicated in mesenchymal differentiation. This is supported by the reported interaction of USP53 with ZO-1/TJP1 and ZO-2/TJP2 tight junction scaffolding proteins in polarized epithelial cells of the ear in mice, where it was suggested that USP53 is part of the tight junction complex^6^. Alternatively, USP53 can act as a decoy protein protecting complexes from other deubiquitinases, as is the case with USP18 regulating TAK1-dependent signaling in a protease-independent manner^29^.

Cantu syndrome (OMIM#:239850) is a rare condition characterized clinically by hypertrichosis (excessive hair growth), cardiomegaly, and bone abnormalities^30^. A 375 kb duplication on chromosome 4q26-27 has been identified in a patient with severe Cantu syndrome combined with obesity (BMI > 40)^27^. The duplication region encompasses the three genes *MYOZ2*, *FABP2*, and *USP53*^27^. Since increased copy number (and presumably increased expression) was reported in the Cantu syndrome patient with multiple bone abnormalities^27^, we first attempted overexpression strategies by transient transfection or infection with constitutive or inducible *Usp53* expression vectors. In all cases, mesenchymal cells did not survive the addition of extraneous *Usp53* coding sequence (data not shown). When *Usp53* expression was decreased using shRNA-mediated knockdown, this affected mesenchymal cell lineage-making decisions.

In the context of mesenchymal differentiation, the depletion of either *Usp53* (Figs. 4, 5, and 8) or *Naca* (Supplementary Fig. S3) in ST2 cells enhanced osteoblastogenesis and inhibited adipogenesis. This similar biological outcome provides a link between the transcriptional regulation of *Usp53* and its functional characterization in regards to mesenchymal lineage selection. The data provide strong circumstantial evidence that NACA and USP53 form part of a common signaling pathway regulating the differentiation of mesenchymal progenitors. Another approach that could be used to support this interpretation is to alter the gene dosage of *Usp53* and *Naca* in compound heterozygous mutant mice in vivo to confirm the contribution of the pathway to mesenchymal commitment and differentiation.

We show that reducing *Usp53* expression modulates lineage selection in BMSCs and established bipotential ST2 stromal cells, favoring osteoblastogenesis. This suggests that USP53 normally acts to block osteoblast differentiation. It appears counter-intuitive that the induction of the expression of an anti-osteoblastogenic gene could contribute to the mechanisms propagating the anabolic effect of iPTH. This paradox may be due to a dosage effect, as neither of the shRNAs directed at *Usp53* were able to completely abrogate USP53 expression (causing between 50 and 70% inhibition), although this seems unlikely. Another possibility is that the effect of USP53 on lineage selection may be influenced in vivo due to the niche environment of the stem/stromal cells^31,32^. During osteoblastogenesis, the impact of *Usp53* knockdown on PTH responses was different depending on the maturation stage of the cells (compare Fig. 7B, E). One interpretation of this result is that the effect of USP53 downstream from iPTH is mediated by a differentiated cell type instead of through stem cells or early lineage cells. Osteocytes, which are critical for maximal iPTH anabolic responses^13^, represent a probable target. Finally, it remains possible that USP53 is required for the endocrine role of PTH in regulating mineral homeostasis and not for its osteoanabolic effect. Answers to these questions will be provided by phenotype characterization following inactivation of *Usp53* using a floxed *Usp53* allele and differentiation stage-specific *Cre* drivers.

This is the first report characterizing the regulation and function of *Usp53 *in vitro. In summary, we show that *Usp53* expression is induced by the PTH-activated NACA axis in osteoblasts. We also offer a mechanism involving the NACA-JUN-CREB trio regulating *Usp53* transcription. Our data strongly suggest that *Usp53* regulates the lineage fate of mesenchymal cells by impacting osteoblastogenesis and adipogenesis. At the pathological level, increased marrow fat content is observed in bone loss conditions such as age-related osteoporosis^33–35^. It will prove interesting to inactivate *Usp53* in specific cell types in vivo and uncover the molecular mechanism by which USP53 alters mesenchymal differentiation and function to mediate PTH responses.

## Materials and methods

### Plasmids, antibodies, and reagents

The wildtype-NACA, NACA-S99D mutant^14^, and NACA delta DBD (69–80) mutant^16^ expressing vectors were previously described. The PKA-dominant-negative (DN-PKA) (cAMP-unresponsive mutant of R1α regulatory subunit) expression vector pcDNA3.1-DN-PKA is a gift provided by N. O. Dulin (University of Chicago, Chicago, IL)^23^. The affinity-purified chicken anti-NACA^15^, the rabbit pan-specific anti-NACA antibody^18,19^, and the rabbit polyclonal anti-NACA-phosphoS99 (anti-pS99)^14^ antibodies have also been previously reported. Rabbit anti-USP53 (HPA035844, Prestige antibodies) was purchased from Sigma-Aldrich. Rabbit anti-GAPDH (HRP conjugate, catalog no. 8884) was purchased from Cell Signaling. PTH(1–34) was purchased from Bachem (Torrance, CA) and N^6^-benzoyladenosine cAMP (6Bnz-cAMP; catalog no. B009) was from Biolog (Bremen, Germany). Ascorbic acid, β-glycerophosphate, dexamethasone (Dex), 3-isobutyl-1-methylxanthine (IBMX), insulin, and hexadimethrine bromide were obtained from Sigma-Aldrich.

### Primary cultures and cell lines

ST2 stromal cells (obtained from Riken Cell bank, Tsukuba, Japan) and MC3T3-E1 (subclone 4) osteoblastic cells (obtained from ATCC, Manassas, VA) were grown in minimum essential medium alpha (α-MEM, Gibco) supplemented with 10% fetal bovine serum (FBS, Hyclone, Logan, UT), 100 U/ml penicillin, and 100 µg/ml streptomycin sulfate at 37 °C in a humidified atmosphere with 5% CO2. The HEK-293 T cell line (ATCC) was cultured in Dulbecco’s modified Eagle’s medium (DMEM, Gibco) supplemented with 10% FBS under the same conditions.

Murine BMSCs were isolated from 6-week-old C57BL/6 male mice (obtained from Charles River Canada Laboratories) as previously described^36^. Briefly, tibia and femoral marrow compartments were used to isolate BMSCs. After cutting the epiphysis, the femurs and tibia were flushed with complete media using a 27-gauge needle. The flushed cells were then filtered using a 70 µm filter mesh to get rid of bone chips and cell clumps. Finally, the cells were resuspended in additional complete medium and cultured in DMEM supplemented with 20% FBS, 100 U/ml penicillin, and 100 µg/ml streptomycin sulfate for differentiation experiments. Neonatal murine calvarial cells were harvested from calvariae of 3-day-old WT C57BL/6 male mice. Primary osteoblasts were isolated as previously described^14^.

### Chip Sequencing (ChIP-Seq), RNA-Sequencing (RNA-Seq), and Chromatin immunoprecipitation (ChIP)

Conventional ChIP assay, ChIP-Seq, and RNA-Seq were performed as previously described^18^. The ChIP-Seq against NACA has identified a peak in Chromosome 3 (3qG1); Chr3: 122,984,707-122,984,915. For ChIP-PCR, *Usp53* promoter fragment was amplified using 5′-CTGAAACCCACAGAACCCCG-3′ forward and 5′-GGATTTCCACGGCAGACGC-3′ reverse primers. Reactions were performed using Power SYBR Green master mix following the manufacturer's instructions (ThermoFisher Scientific) and carried out in a QuantStudio 7 Flex Real-Time PCR System (ThermoFisher Scientific). For RNA-Seq, a cutoff of 1 in logscale was used to filter regulated genes by a twofold change. David Bioinformatics Resources 6.8 (NIAID,NIH) was used for gene ontology (GO) analysis and functional clustering ^37^.

The ChIP-Seq and RNA-Seq data were deposited in the Gene Expression Omnibus database of the National Institute for Biotechnology Information (Accession numbers: GSE147070 and GSE154355, respectively).

### Electrophoresis mobility shift assay (EMSA)

EMSA assays were performed as described elsewhere^18^. HEK-293T cells were transfected with 10 µg of NACA-expressing vector^14^. Twenty-four hours (24 h) following transfection, cells were treated for 1 h with 100 μm 6Bnz-cAMP to induce phosphorylation of NACA at residue Serine 99 and nuclear translocation^14^. Oligonucleotide probes spanning the NACA binding site (NACAbds) within the *Usp53* promoter region (5′-ACCCCGGCTCAGATCCCGCG-3′) or the positive control NACAbds from the *Myoglobin* promoter region (5′-AGGGCCAGAGAAAGACA-3′)^20^ were synthesized with a GG overhang. The probes were ^32^P-labeled using [α^32^P]-dCTP (PerkinElmer, Waltham, MA) and Klenow DNA Polymerase I (New England Biolabs, Ipswich, MA).

### Luciferase assay

Transfections and luciferase assays were performed as previously described ^19^. MC3T3-E1 cells were seeded at a density of 20,000 cells/well, 14 h prior to transfection. In each well, 300 ng of *Usp53* promoter reporter vector and 15 ng of Renilla luciferase internal control vector (pRL-null, Promega) were transfected using the lipofectamine LTX and PLUS Reagent kit following the manufacturer’s instructions (Invitrogen). When indicated, 100 ng of NACA-S99D mutant or NACA delta DBD (69–80) mutant and 50 ng of CREB-expressing vector (MR204788, Origene), 50 ng of JUN-expressing vector^22^ or 100 ng of DN-PKA expressing vector were co-transfected. Twenty-four (24) h post transfection, the Dual-Glo luciferase assay was performed according to the manufacturer’s protocol (Promega) and signal was measured using a VICTOR Nivo multimode microplate reader (Perkin Elmer, Waltham, MA). Results were calculated by normalizing Firefly relative light units to Renilla relative light units and are expressed as fold-change over the empty vector. When indicated, cells were treated with 100 nM PTH(1–34) for 24 h before luciferase measurement.

### Vector cloning

Promoter cloning was performed as previously described^19^. Briefly, genomic DNA from MC3T3-E1 cells was used to amplify the *Usp53* promoter fragment (-2325/+ 238 bp). The fragment was subcloned into the pGL4.10[LUC2] vector (Promega, Madison, WI) using KpnI and NheI restriction sites that were added during the PCR step. All generated vectors were verified by sequencing. Primer sequences and plasmids are available upon request.

### Short hairpin RNA (shRNA) gene silencing

Vectors expressing the short hairpins RNAs (shRNAs) targeting *Naca* were generated as previously described^18^. Vectors expressing shRNAs targeting *Usp53* (shUsp53, TRC no. 0000030914; shUsp53.1, TRC no. 0000030918) and the non-targeted control shRNA (shScr; #SHC016) were purchased from Sigma-Aldrich. For stable silencing of *Naca* and *Usp53*, lentiviral infections were performed as previously described^18,19^. Briefly, lentiviral particles were prepared by transfecting HEK-293 T cells with 10 µg of shRNA expressing vector, 7.5 µg psPAX2 (viral packaging vector, a gift from Dr. Didier Trono; Addgene plasmid #12260), and 2.5 µg pMD2.G (viral envelope encoding vector, a gift from Dr. Didier Trono; Addgene plasmid #12259), using the calcium/phosphate DNA-precipitation technique^38^. Lentivirus-enriched medium was collected 48 h post-transfection, filtered using a 0.45 µm filter (Millipore, Billerica, MA), and supplemented with 8 µg/ml hexadimethrine bromide (Polybrene, Sigma-Aldrich). The lentiviral medium was used to infect BMSCs, ST2 or MC3T3-E1 cells for 24 h. Infected cells were then cultured in the presence of 2.5 µg/ml (BMSCs and ST2) or 5 µg/ml (MC3T3-E1) of puromycin for selection. Selection was performed for 5 days.

### Osteogenic differentiation

ST2 cells or MC3T3-E1 cells were plated at 50,000 cells/cm^2^ for differentiation assays. To induce osteogenic differentiation of ST2 cells, cells were incubated in α-MEM medium at 37 °C with the supplementation of 50 µg/ml of ascorbic acid and 100 ng/ml of BMP2 (Gibco). For osteogenic differentiation, MC3T3-E1 cells were cultured in α-MEM medium at 37 °C with the supplementation of 50 µg/ml of ascorbic acid and 10 mM of β-glycerophosphate. Medium was changed every 2 days for the duration of the experiment. When indicated, cultures were treated with 100 nM of PTH(1–34) for 2 h prior to analysis.

### Adipogenic differentiation

ST2 cells were plated at 50,000 cells/cm^2^ for adipogenic differentiation. The cells were incubated in DMEM medium at 37 °C with IBMX (0.5 mM), dexamethasone (1 µM), insulin (1 µg/ml), and rosiglitazone (1 µM) for the first day. On day 2, differentiation medium was changed and supplemented with only insulin (1 µg/ml) and rosiglitazone (1 µM). Complete DMEM medium (without supplements) was used from day 4 until the end of the differentiation assay on day 6. When indicated, cultures were treated with 100 nM of PTH(1–34) for 2 h prior to analysis.

### RNA isolation and quantitative real-time PCR

Total RNA was extracted from cells or tissues using TRIzol reagent following the manufacturer’s instructions (Invitrogen). 1–2 µg of RNA were reverse-transcribed into cDNA using the high-capacity cDNA reverse transcription kit (Thermo Fisher Scientific), following the manufacturer’s protocol. Gene expression was assessed using the Taqman Fast Advanced Master Mix (Thermo Fisher Scientific) and the following TaqMan primers: *Usp53* (Mm00476778-m1), *Gapdh* (Mm99999915-g1), *Dlx5* (Mm00438430), *Alpl* (Mm00475834-m1), *Sp7* (Mm00504574-m1), *Bglap2* (AIWR1XJ), *Fabp4* (Mm00445878-m1), *Cebpβ* (Mm00843434-s1), *Cebpα* (Mm00514283-s1), *Pparg2* (Mm00440940-m1), and *AdipoQ* (Mm00456425-m1). Expression levels of *Usp53* (long and short isoforms) were assessed using the Power SYBR Green PCR Master Mix (Thermo Fisher Scientific) and the following isoform-specific , oligonucleotide primers: *Usp53* short isoform, forward, 5′-GAAGTGTCCTAGTAACTGTGGCC-3′ and reverse, 5′-GAATGAAAGCAACTGTGATACCCC-3′; *Usp53* long isoform, forward, 5′-CGACACAGGGATTTGGTTGATG-3′, and reverse, 5′-CAGAGGTGTAGCTCTCATGGG-3′; and *Gapdh*, forward, 5′-CATCACTGCCACCCAGAAGACTG-3′, and reverse, 5′-ATGCCAGTGAGCTTCCCGTTCAG-3′. All reactions were carried out in a 7500 real-time PCR system (Thermo Fisher Scientific).

### Staining

Alkaline phosphatase (ALP) staining was performed on ST2 cells on day 12 of osteogenic differentiation. Staining was performed using the TRACP and ALP double-stain kit (TAKARA) following the manufacturer’s instructions. Von Kossa staining was performed on ST2 cells on day 8 of osteogenic differentiation. Cells were fixed in 10% formalin at RT and stained for mineralized matrix using 5% silver nitrate. Alizarin red staining of MC3T3-E1 cells was performed as previously described ^19^. Alizarin red staining was performed on MC3T3-E1 cells on day 21 of osteogenic differentiation. Briefly, cells were fixed in 70% ethanol for 1 h at − 20 °C and then incubated with alizarin red solution (40 mM, pH 4.2) (Sigma-Aldrich) for 15 min at RT. Cells were then washed with dH_2_O to remove excess stain. Finally, cells were washed with PBS (1x) for 15 min and photographed. ALP, Von Kossa, and alizarin red staining were quantified using ImageJ (IJ1.46r, NIH) and presented as mean percentage (± SD) of stained area / total area of 3 independent experiments (n = 3). Oil Red O staining was performed on ST2 cells on day 6 of adipogenic differentiation. Cells were fixed in 10% formalin (Sigma-Aldrich) for 45 min at RT. Following fixation, cells were rinsed with dH_2_O and then 60% isopropanol for 2–5 min. Cells were then incubated with Oil Red O solution for 5 min. Finally, cells were rinsed 3–4 times with dH_2_O and photographed. For Oil Red O quantification, 150 µl of 100% 2-propanol was added to each well and incubated for 5 min at room temperature. Optical density (OD) was then measured at 520 nm using a VICTOR Nivo multimode microplate reader (Perkin Elmer, Waltham, MA). Data was presented as mean of OD (± SD) of 3 independent experiments (n = 3).

### In vivo osteogenesis assay

Animal experimentation was carried out in compliance with the ARRIVE guidelines. All animal procedures were reviewed and approved by the McGill Institutional Animal Care and Use Committee and followed the guidelines of the Canadian Council on Animal Care. For the in vivo osteogenesis assay, 2 × 10^6^ shScr or shUsp53-knockdown BMSCs were seeded in Gelfoam absorbable collagen sponges (Pfizer) and implanted subcutaneously on the backs of 6-wk-old athymic nude mice (Crl:NIH-Lystbg Foxn1nu Btkxid; 201; Charles River Laboratories). After 4 weeks, the transplants (n = 4 for both shScr and shUsp53) were harvested and further processed for micro-CT and histological analysis. For micro-CT, transplants were fixed in 4% PFA in PBS for 48 h at room temperature and washed with 1 × phosphate-buffered saline. Transplants were then scanned with a 6-µm pixel size using a SkyScan 1272 µCT system (Bruker, Belgium). The parameters were 5 µm pixel size, 50 kV, 194 µA, 0.5 mm Al filter, angular rotation step of 0.450, and an exposure time of 500 ms with a total scan duration of 45 min. Three-frame averaging was used to improve the signal-to-noise ratio. After scanning, 3-dimensional (3D) microstructural image data were reconstructed using the manufacturer’s software (SkyScan NRecon). For histology, transplants were then decalcified in EDTA and embedded in paraffin. Paraffin blocks were sectioned (6 µm) and stained with H&E or incubated with specific primary antibodies against Osterix (1/100; ab22552; Abcam) or Perilipin (1/200; ab3526; Abcam). Prior to immunostaining, sections were pretreated with citrate buffer (pH 6.0) and heated for 10 min at a temperature between 95 °C and 100 °C for antigen retrieval. Primary antibodies were detected using Alexa Fluor 594 conjugated goat anti-rabbit IgG (H + L) (1/250; Invitrogen). Nuclei were stained with ProLong Gold antifade reagent with DAPI (Thermo Fisher Scientific). Images were acquired using a Leica DMR fluorescence microscope (Leica Microsystems, Wetzlar, Germany) connected to a digital DP70 camera (Olympus, Center Valley, PA). Image processing included whole image channel filtering to remove noise and whole image adjustment of brightness, contrast, color balance, and sharpening using Adobe Photoshop v. 12.1. Photoshop images were then flattened and imported into Adobe Illustrator v. 15.1 to build the final montages.

### Western blotting

Protein extraction and immunoblotting were performed as previously described^19^. Whole cell extracts were prepared from MC3T3-E1, ST2, and BMSC cells. Cells were washed twice with ice-cold PBS (1x) and scraped with lysis buffer (300 mM NaCl, 50 mM HEPES, pH 7.6, 1% Triton X-100, and protease/phosphatase inhibitor cocktail (Cell Signaling)). Cells were then kept on ice for 10 min and briefly sonicated with 1 or 2 pulses for 15 s using an Ultrasonic dismembrator model 500 (Thermo Fisher Scientific, Waltham, MA). Following sonication, cells were spun down at 4 °C for 15 min at maximum speed. Supernatant was aliquoted and stored at − 80 °C. For western blots, equal amounts of cell extracts (10 µg protein) were resuspended in 6X Laemmli buffer. The samples were boiled for 5 min and run on a denaturing SDS‐PAGE for 1.5 h then transferred to a PVDF membrane (Amersham, UK). The membrane was blocked for 45 min in 5% non-fat dry milk. After blocking, the membrane was incubated with the primary antibody diluted 1:1000 in 5% non-fat dry milk, overnight at 4 °C. The membrane was later incubated with the secondary antibody conjugated with horseradish‐peroxidase, anti‐mouse, or anti‐rabbit‐ HRP, diluted 1:20,000. Development was done using the Western Lightening Chemiluminescence Kit (Perkin Elmer, Akron, OH, USA) and exposure to X-Ray film. The protein bands were visualized on the developed film. Western blot analysis and quantification was performed with ImageJ (IJ1.46r, NIH). Uncropped original blots are determined to be too large to place in multi-panel figures but are presented in full-length in Supplementary Figures S4–S8.

### Statistical significance

Data are presented as means ± one standard deviation (SD). Comparisons were made by analysis of variance (ANOVA) with Bonferroni’s *post* *hoc* test , or unpaired *t* tests. A probability (P) value lower than 0.05 was accepted as significant. For each panel, three independent experiments were performed, each in triplicates.

## Supplementary Information

Supplementary Information.
