# Supplementary material for: Ubiquitin specific peptidase Usp53 regulates osteoblast versus adipocyte lineage commitment
Source: Sci Rep. 2021 Apr 19;11:8418. doi: 10.1038/s41598-021-87608-x (PMC8055676; doi:10.1038/s41598-021-87608-x)
Supplement: Supplementary file 1 — Supplementary Information. [file 41598_2021_87608_MOESM1_ESM.pdf]

Ubiquitin specific protease *Usp53* regulates osteoblast versus adipocyte lineage commitment

Hadla Hariri[a,b], William N. Addison[a] and René St-Arnaud[a-d#]

[a]Research Centre, Shriners Hospital for Children – Canada, Montreal (Quebec) Canada
[b]Department of Human Genetics, McGill University, Montreal, (Quebec) Canada
[c]Department of Surgery, McGill University, Montreal, (Quebec) Canada
[d]Department of Medicine, McGill University, Montreal, (Quebec) Canada

#Address Correspondence to: René St-Arnaud
Research Centre
Shriners Hospital for Children – Canada
1003 Decarie Boulevard
Montreal (Quebec) Canada H4A 0A9

Tel: (514) 282-7155
Fax: (514) 842-5581
rst-arnaud@shriners.mcgill.ca

Running Title: *Usp53* in mesenchymal lineage selection

A

Gene	NACA motif	Distance to TSS	RNA-Seq	Reference
Bglap2	GCACAGAGTAG (P)<br>GCACACAGTAG (D)	-35/-45 bp (P)<br>-250/-260 bp (D)	Upregulated by PTH	*Akhouayri et al., 2005
Lrp6	GCAGAAAGTA (P)<br>GCACAAAGAG (D)	-340/-331 bp (P)<br>-376/-367 bp (D)	Upregulated by PTH	Pelicelli et al., 2018
Nfil3	CGGCCAGAGAAGG	-165/-177 bp	Upregulated by PTH	Hariri et al., 2020
Usp53	GGCTCAGATCCCC	-333/-345 bp	Upregulated by PTH	This manuscript
Isy1	GGAGAGAATTG	-24/-35 bp	Upregulated by PTH	Unpublished
Dnajb4	GCACAGCCCGA**	-7/-18 bp	Upregulated by PTH	Unpublished
Relb	GCAGAAAACTG**	-736/-747 bp	Upregulated by PTH	Unpublished
Bpnt1	CCAGAAACCAG**	-127/-138 bp	Upregulated by PTH	Unpublished

* The NACA response element in the *Bglap2* promoter was previously characterized by Akhouayri et al., 2005.
** False-positive targets identified by ChIP-Seq but could not be validated by conventional ChIP assays.

B

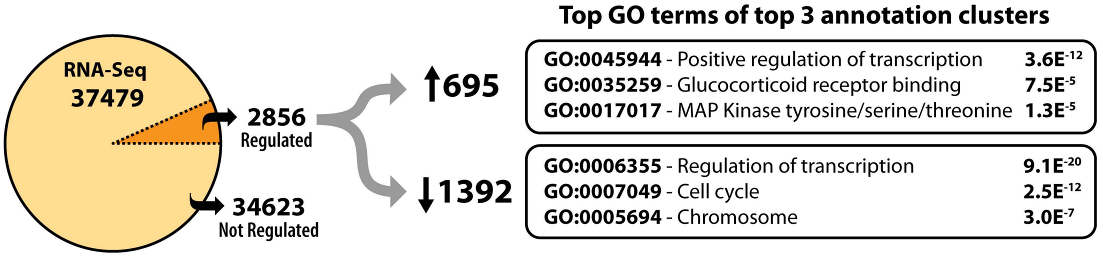

C

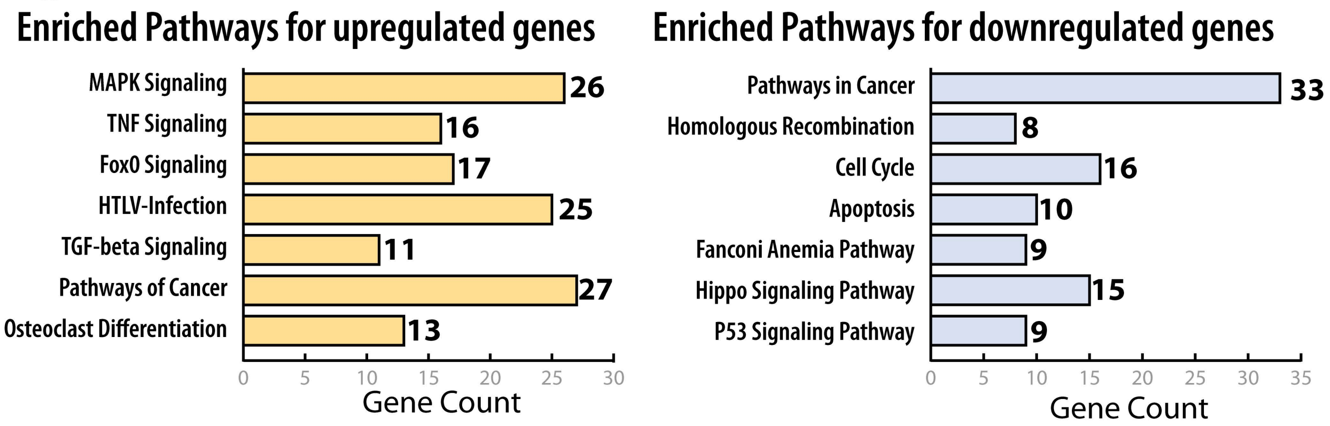

Supplementary Figure S1. ChIP-Seq and RNA-Seq analysis

(A) A table of the NACA-ChIP-Seq peaks identified in MC3T3-E1 cells treated for 30 min with vehicle or 100nM PTH(1-34). The proximal region of the promoters was analyzed and transcription start sites (TSS) were determined in IGV. NACA binding motifs were determined by Mat Inspector (Genomatix software suite). Transcriptome analysis by RNA-Seq of MC3T3-E1 cells treated for 1h with vehicle or PTH(1-34). (B) A pie chart of genes either regulated or not regulated by PTH(1-34) treatment. Genes were classified as regulated if their expression levels are changed by 2-folds or more. The top GO terms of top 3 annotation clusters from DAVID are shown with corresponding *P* values. (C) The Kyoto Encyclopedia of Genes and Genomes (KEGG) enriched pathways from DAVID for up-regulated and down-regulated genes. (P), proximal; (D), distal. Panels B and C were created with BioRender .com.

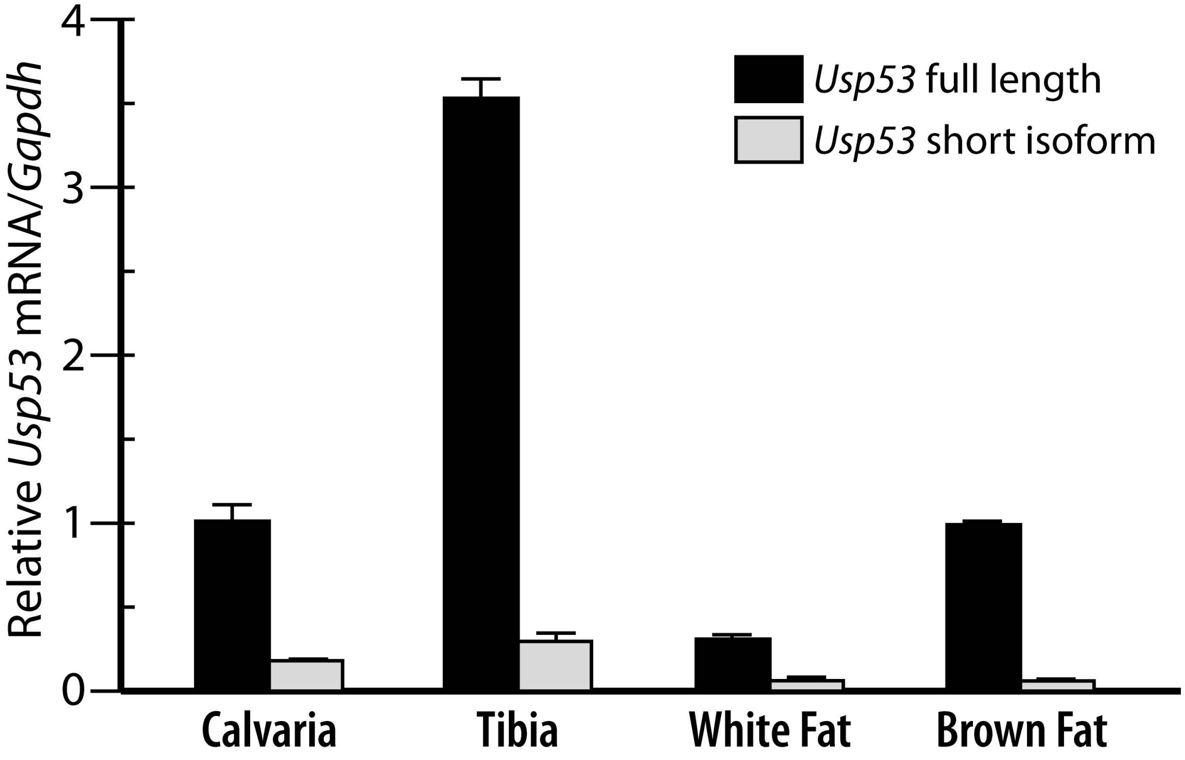

Supplementary Figure S2. *Usp53* long isoform is predominant in skeletal tissues

Gene expression analysis of *Usp53* expression in different skeletal tissues of wildtype C57BL/6 mice (n=4). Quantitative RT-qPCR using isoform-specific primers against *Usp53*, normalized to *Gapdh*, was performed on RNA isolated from long and flat bones as well as gonadal (white fat) and interscapular (brown fat). Results are presented as means ± SD.

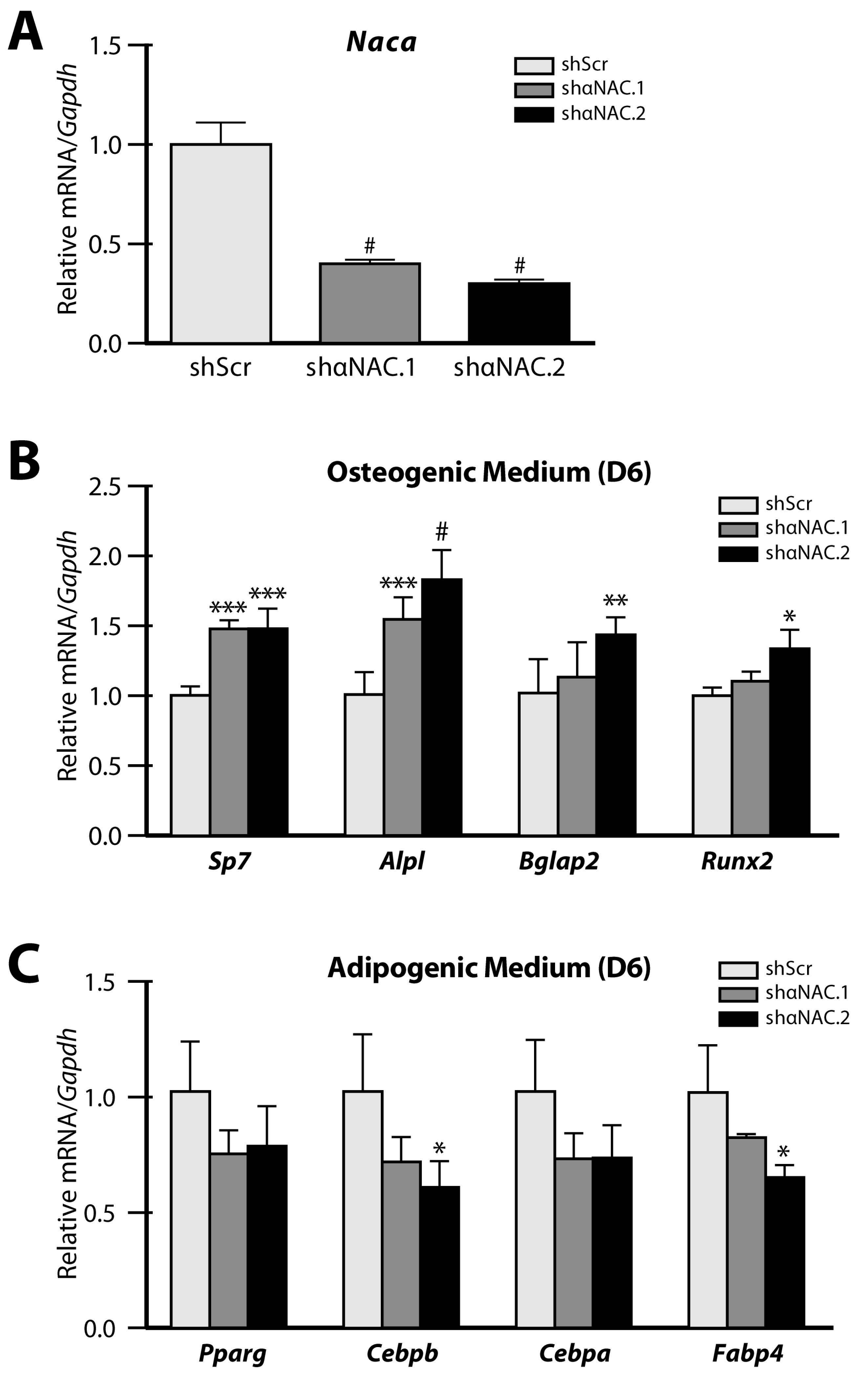

Supplementary Figure S3. ***Naca*** **modulates osteogenic and adipogenic differentiation *in vitro***

(A) Knockdown of *Naca* in ST2 cells. Quantitative RT-qPCR using a probe against *Naca,* normalized to *Gapdh*, was performed on RNA isolated from shScr-stably transfected or shNaca-stably transfected pools of ST2 cells at D0. Gene expression analysis of osteogenic (B) and adipogenic (C) differentiation markers at D6. Quantitative RT-qPCR using TaqMan probes against osteoblast or adipocyte differentiation markers, normalized to *Gapdh,* on RNA isolated from ST2 cells expressing scrambled shRNAs (shScr) targeting control and shRNAs (shαNAC.1 and shαNAC.2) targeting *Naca*, cultured under osteogenic or adipogenic conditions for 6 days. Results are presented as the mean fold change ± SD. *, P≤0.05; **, P≤0.01; ***, P≤0.001; #, P<0.0001; ANOVA with Bonferroni's post-hoc test for all panels.

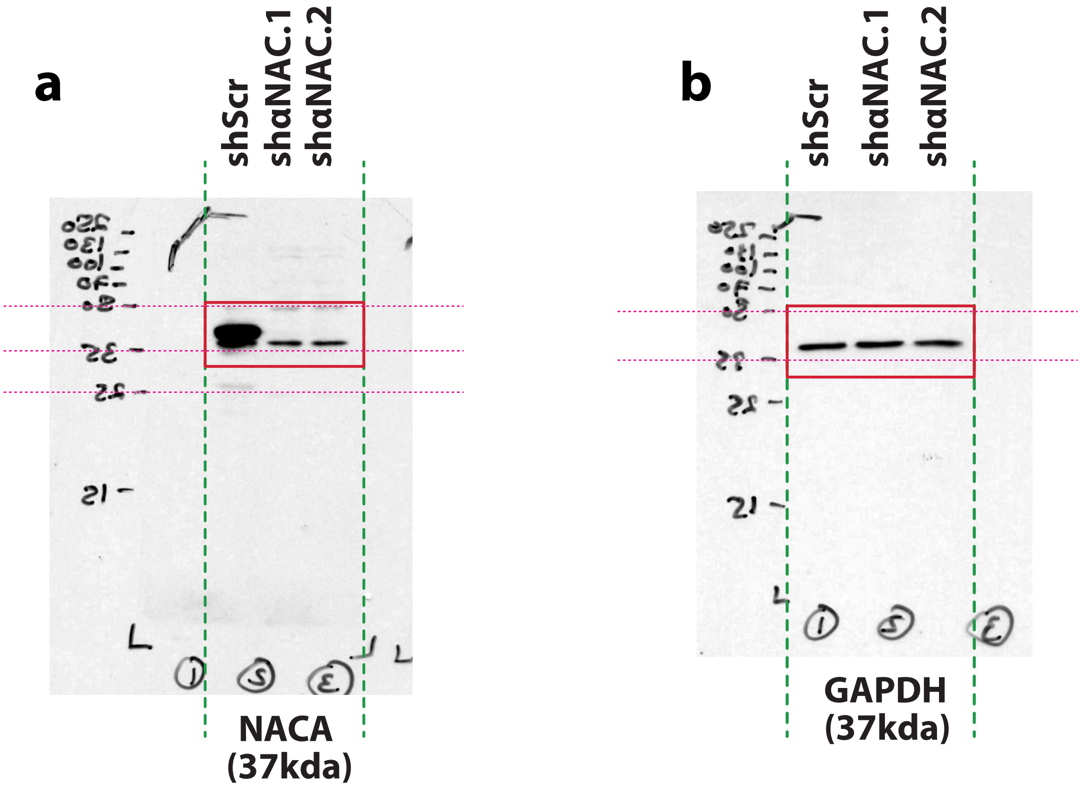

Supplementary Figure S4. Raw western blots corresponding to the cropped blots shown in Figure1F

Western blotting (a) of NACA protein from whole cell extracts of MC3T3-E1 cells stably expressing shRNAs targeting *Naca* (shαNAC.1 and shαNAC.2) and scrambled shRNA (shScr) as control. (b) The same membrane was subsequently stripped and blotted against GAPDH for loading control. The red box represents the cropped piece of the blot presented in figure 1F in the manuscript. Dotted red lines were used to better define the molecular weight markers (Kda).

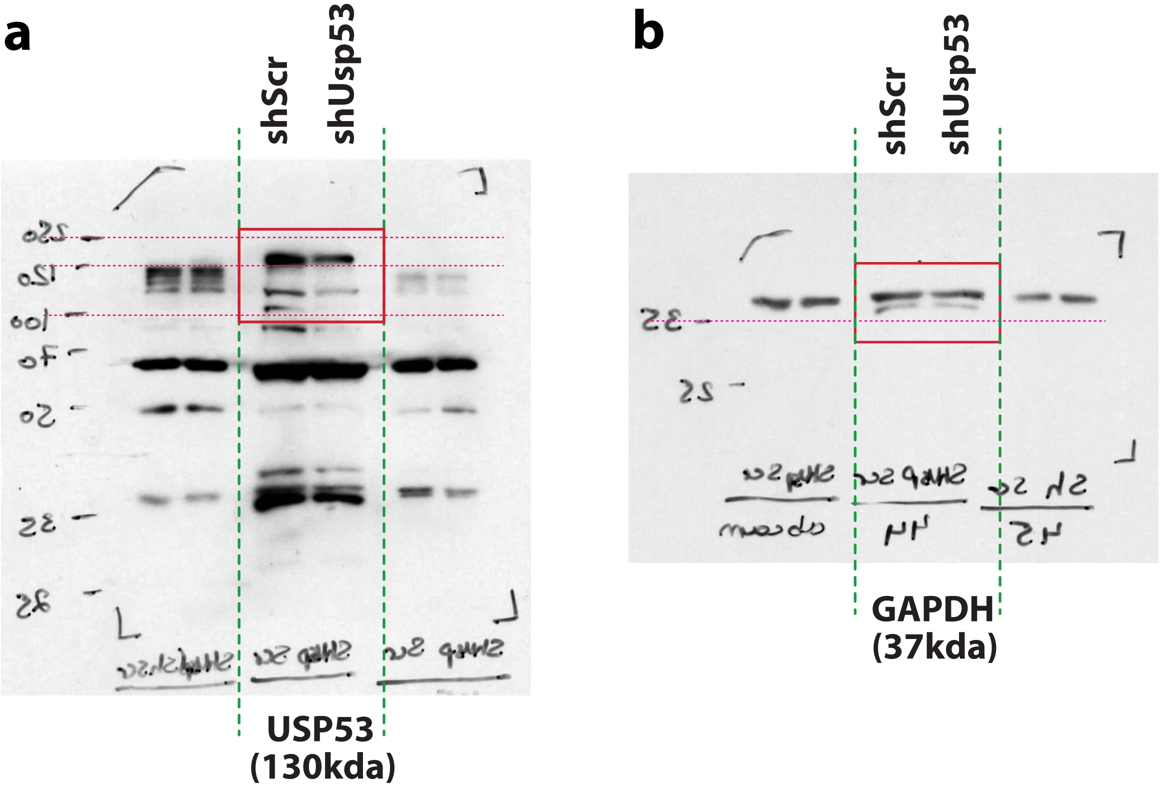

Supplementary Figure S5. Raw western blot data corresponding to the cropped blots shown in Figure 4C

Western blotting (a) of USP53 protein from whole cell extracts of ST2 cells stably expressing shRNAs targeting *Usp53* (shUsp53) and scrambled shRNA (shScr) as control. (b) The same membrane was subsequently cut, stripped, and blotted against GAPDH for loading control. The red box represents the cropped piece of the blot presented in figure 4C in the manuscript. Dotted red lines were used to better define the molecular weight markers (Kda).

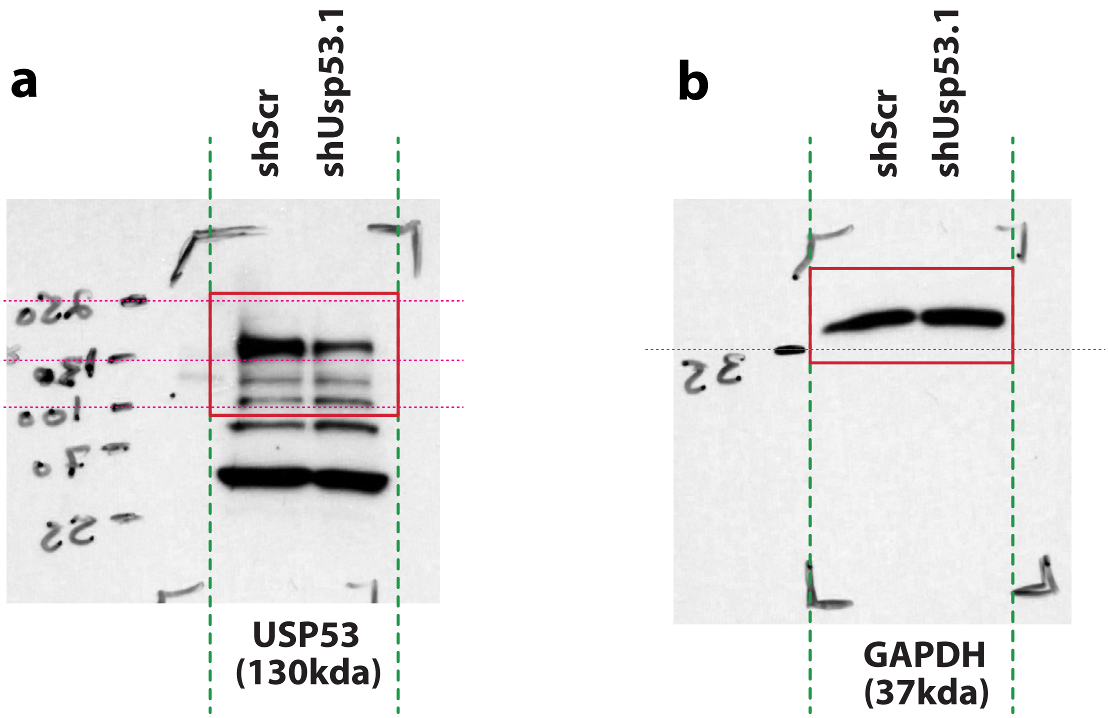

Supplementary Figure S6. Raw western blot data corresponding to the cropped blots shown in Figure 5C

Membrane was cut prior to hybridization with different antibodies (anti-USP53 and anti-GAPDH-HRP conjugated antibodies).
Western blotting (a) of USP53 protein from whole cell extracts of ST2 cells stably expressing shRNAs targeting *Usp53* (shUsp53.1) and scrambled shRNA (shScr) as control. (b) Western blot blotted against GAPDH for loading control. The red box represents the cropped piece of the blot presented in figure 5C in the manuscript. Dotted red lines were used to better define the molecular weight markers (Kda).

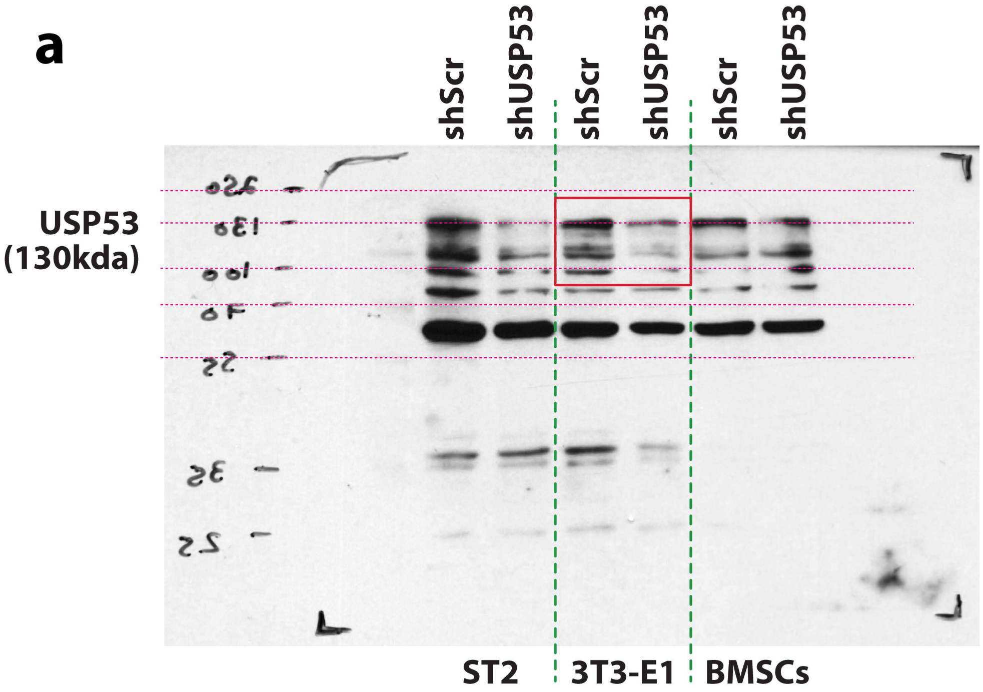

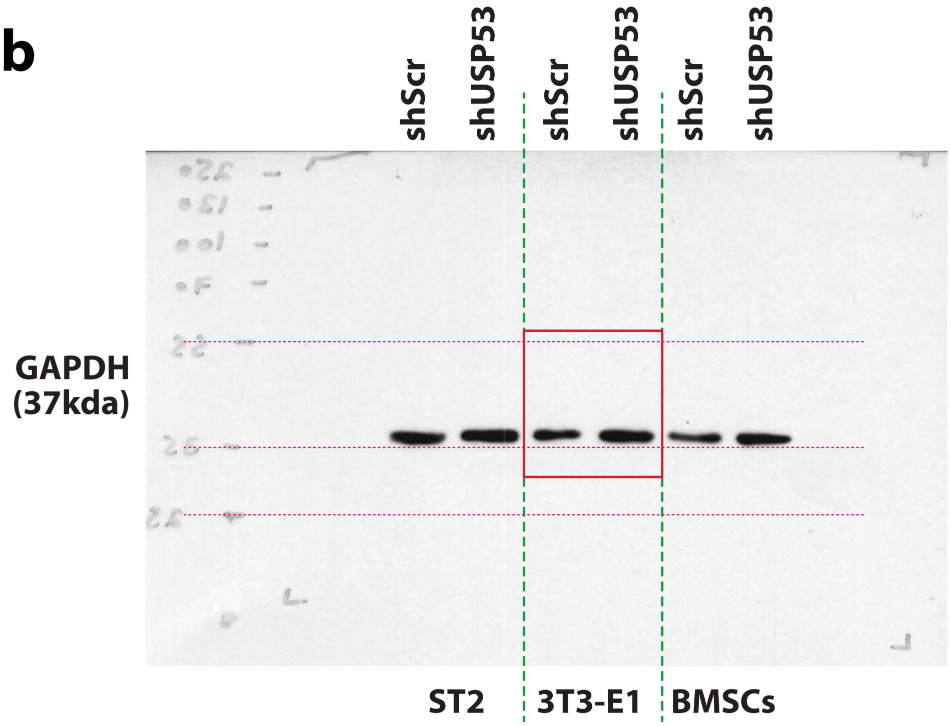

Supplementary Figure S7. Raw western blot data corresponding to the cropped blots shown in Figure 6B

Western blotting (a) of USP53 protein from whole cell extracts of ST2, MC3T3-E1, and BMSCs stably expressing shRNAs targeting *Usp53* (shUsp53) and scrambled shRNA (shScr) as control. (b) The same membrane was subsequently stripped and blotted against GAPDH for loading control. The red box represents the cropped piece of the blot presented in figure 6B in the manuscript. Dotted red lines were used to better define the molecular weight markers (Kda).

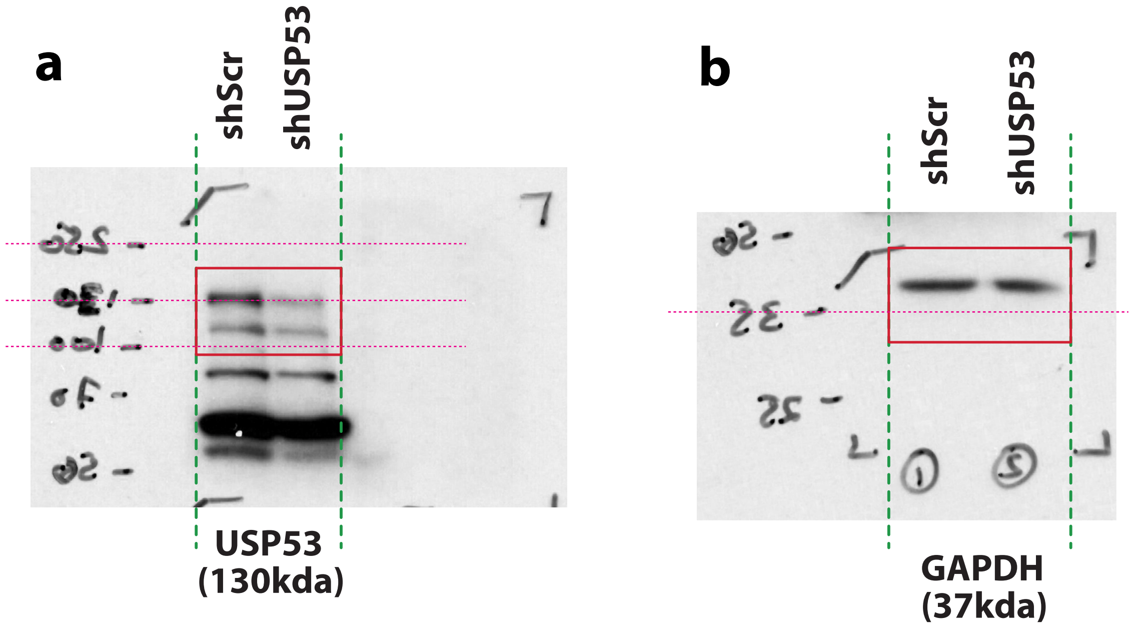

Supplementary Figure S8. Raw western blot data corresponding to the cropped blots shown in Figure 8C

Membrane was cut prior to hybridization with different antibodies (anti-USP53 and anti-GAPDH-HRP conjugated antibodies).
Western blotting (a) of USP53 protein from whole cell extracts of ST2 cells stably expressing shRNAs targeting *Usp53* (shUsp53.1) and scrambled shRNA (shScr) as control. (b) Western blot blotted against GAPDH for loading control. The red box represents the cropped piece of the blot presented in figure 8C in the manuscript. Dotted red lines were used to better define the molecular weight markers (Kda).
